# Molecular machines operating on the nanoscale: from classical to quantum

**DOI:** 10.3762/bjnano.7.31

**Published:** 2016-03-03

**Authors:** Igor Goychuk

**Affiliations:** 1 Institute for Physics and Astronomy, University of Potsdam, Karl-Liebknecht-Str. 24/25, 14476 Potsdam-Golm, Germany

**Keywords:** anomalous dynamics with memory, Brownian nanomachines, nanoscale friction and thermal noise, quantum effects, thermodynamic efficiency

## Abstract

The main physical features and operating principles of isothermal nanomachines in the microworld, common to both classical and quantum machines, are reviewed. Special attention is paid to the dual, constructive role of dissipation and thermal fluctuations, the fluctuation–dissipation theorem, heat losses and free energy transduction, thermodynamic efficiency, and thermodynamic efficiency at maximum power. Several basic models are considered and discussed to highlight generic physical features. This work examines some common fallacies that continue to plague the literature. In particular, the erroneous beliefs that one should minimize friction and lower the temperature for high performance of Brownian machines, and that the thermodynamic efficiency at maximum power cannot exceed one-half are discussed. The emerging topic of anomalous molecular motors operating subdiffusively but very efficiently in the viscoelastic environment of living cells is also discussed.

## Introduction

A myriad of minuscule molecular nanomotors (not visible in standard, classical, optical microscopes) operate in living cells and perform various tasks. These utilize metabolic energy, for example, the energy stored in ATP molecules maintained at out-of-equilibrium concentrations, or in nonequilibrium ion concentrations across biological membranes. Conversely, they may replenish the reserves of metabolic energy using other sources of energy, for example, light by plants, or energy of covalent bonds of various food molecules by animals [1]. The main physical principles of their operation are more or less understood by now [2–3], although the statistico-mechanical details of any single particular molecular motor (e.g., a representative of a large family of kinesin motors) are not well understood.

The advances and perspectives of nanotechnology have inspired us to devise our own nanomotors [4–6]. Learning from nature can help to make the artificial nanomotors more efficient, and possibly even better than those found in nature. Along this way, understanding the main physical operating principles within the simplest, minimalist physical models can indeed be of help.

First of all, any periodically operating motor or engine requires a working body undergoing cyclic changes and a source of energy to drive such cyclic changes. Furthermore, it should be capable of doing work on external bodies. In the case of thermal heat engines, the source of energy is provided by heat exchange with two heat reservoirs or baths at different temperatures, *T*_1_, and *T*_2 _*> T*_1_, with the maximum possible Carnot efficiency of η*_C_* = 1 − *T*_1_/*T*_2_ [7]. This very famous textbook result of classical thermodynamics (or rather thermostatics) is modified when the heat flow is considered as a function of time. Thus, for an infinitesimally slow heat flow occurring over a finite time, one obtains the Curzon and Ahlborn result, 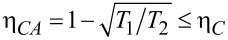
[7–8]. The analogy with heat engines is, however, rather misleading for isothermal engines operating at the same temperature, *T*_1_ = *T*_2_. Here, the analogy with electrical motors is much more relevant. The analogy becomes almost literal in the case of rotary ATP-synthase [9] or flagellar bacterial motors (the electrical nanomotors of living cells). Here, the energy of a proton electrochemical gradient (an electrochemical rechargeable battery) is used to synthesize ATP molecules out of ADP and the orthophosphate P_i_ (the useful work done), in the case of ATP-synthase, or to produce mechanical motion by flagellar motors [1,3]. An ATP-synthase nanomotor can also operate in reverse [9], and the energy of ATP hydrolysis can be used to pump protons against their electrochemical gradient to recharge the “battery”. These and similar nanomotors can operate at ambient temperature in a highly dissipative environment with nearly 100% thermodynamic efficiency defined as the ratio of useful work done to the input energy spent. This is the first counter-intuitive remarkable feature, which needs to be explained. It is easy to derive this result within the simplest model (see below) for an infinitesimally slow operating motor at zero power. At maximum power at a finite speed, the maximum thermodynamic efficiency within such a model is one-half. This is still believed by many to be the maximum, theoretically possible, thermodynamic efficiency of isothermal motors at maximum power. However, this belief is born from underestimating the role played by thermal fluctuations in nonlinear stochastic dynamics and the role of the fluctuation–dissipation theorem (FDT) on the nano- and microscale. It is generally wrong. It is valid only for some particular dynamics, as clarified below by giving three counter-examples. The presence of strong thermal fluctuations at ambient temperature, playing a constructive and useful role, is a profound physical feature of nanomotors as compared with the macroscopic motors of our everyday experience. It is necessary to understand and to develop an intuition for this fundamental feature. Nanomotors are necessarily Brownian engines, very different from their macroscopic counterparts.

## Review

### Fluctuation–dissipation theorem, the role of thermal fluctuations

Motion in any dissipative environment is necessarily related to the dissipation of energy. Particles experience a frictional force, which in the simplest case of Stokes friction is linearly proportional to the particle velocity with a viscous friction coefficient denoted as η. When the corresponding frictional energy losses are no longer compensated for by an energy supply, the motion will eventually stop. However, this does not happen in microworld for micro- or nanosized particles. Their stochastic Brownian motion can persist forever even at thermal equilibrium. The energy necessary for this is supplied by thermal fluctuations. Therefore, friction and thermal noise are intimately related, which is the physical context of the fluctuation–dissipation theorem [10]. Statistical mechanics allows the development of a coherent picture to rationalize this fundamental feature of Brownian motion.

We start with some generalities that can be easily understood within a standard dynamical approach to Brownian motion that can be traced back to pioneering contributions by Bogolyubov [11], Ford, Kac and Mazur [12–13], and others. Consider a motor particle with mass *M*, coordinate *x*, and momentum *p*. It is subjected to a regular, dynamical force *f*(*x*,*t*), as well as the frictional and stochastically fluctuating forces of the environment. The latter are modeled by an elastic coupling of this particle to a set of *N* harmonic oscillators with masses *m_i_*, coordinates *q_i_*, and momenta *p_i_*. This coupling is of the form 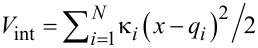, with spring constants κ*_i_*. This is a standard mechanistic model of nonlinear, classical Brownian motion known within quantum dynamics as the Caldeira–Leggett model [14] upon modification of the coupling term or making a canonical transformation [13]. Both classically and quantum mechanically [13] (in the Heisenberg picture) the equations of motion are

[1]
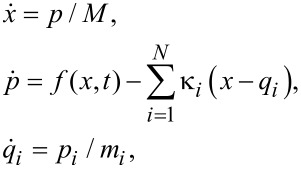


[2]
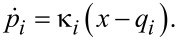


In the quantum case, *x*, *q_i_*, *p*, *p_i_* are operators obeying the commutation relations 
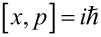
, 
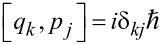
, [*x*,*q_i_*] = 0, [*p*,*p_i_*] = 0. Force, *f*(*x*,*t*), is also operator. Using Green's function of harmonic oscillators, the dynamics of bath oscillators can be excluded (projection of hyper-dimensional dynamics on the (*x*,*p*) plane) and further represented simply by the initial values *q_i_*(0) and *p_i_*(0). This results in a generalized Langevin equation (GLE) for the motor variables

[3]
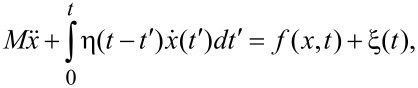


where

[4]
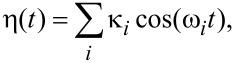


is a memory kernel and

[5]
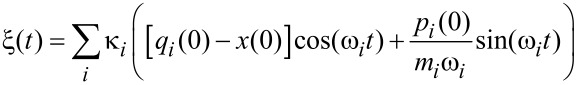


is a bath force, where 
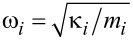
 are the frequencies of the bath oscillators. Equation 3 is still a purely dynamical equation of motion that is exact. The dynamics of [*x*(*t*),*p*(*t*)] is completely time-reversible for any given *q_i_*(0) and *p_i_*(0) by derivation, unless the time-reversibility is dynamically broken by *f*(*x*,*t*) or by boundary conditions. Hence, time-irreversibility within dissipative Langevin dynamics is a statistical effect due to averaging over many trajectories. Such an averaging cannot be undone, i.e., there is no way to restore a single trajectory from their ensemble average. Considering a classical dynamics approach first, we choose initial *q_i_*(0) and *p_i_*(0) from a canonical, hyper-dimensional, Gaussian distribution, ρ(*q_i_*(0),*p_i_*(0)), zero-centered in *p_i_*(0) subspace and centered around *x*(0) in *q_i_*(0) subspace, and characterized by the thermal bath temperature *T*, like in a typical molecular dynamics setup. Then, each ξ(*t*) presents a realization of a stationary, zero-mean, Gaussian stochastic process, which can be completely characterized by its autocorrelation function, 
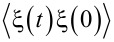
. Here, 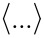 denotes statistical averaging done with ρ(*q_i_*(0),*p_i_*(0)). An elementary calculation yields the fluctuation–dissipation relation (FDR), also named the second FDT by Kubo [10]:

[6]
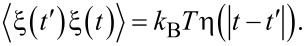


Notice that it is valid even for a thermal bath consisting of a single oscillator. However, a quasi-continuum of oscillators is required for the random force correlations to decay to zero in time. This is necessary for ξ(*t*) to be ergodic in correlations. Kubo obtained this FDT in a very different way, namely by considering the processes of dissipation caused by phenomenological memory friction characterized by the memory kernel η(*t*) (i.e., heat given by the particle to the thermal bath) and absorption of energy from the random force ξ(*t*) (i.e., heat absorbed from the thermal bath). Here, both processes are balanced at thermal equilibrium, and the averaged kinetic energy of the Brownian particle is *k*_B_*T*/2. This is in accordance with the equipartition theorem in classical equilibrium statistical mechanics. This is a very important point. At thermal equilibrium, the net heat exchange between the motor and its environment is zero for arbitrarily strong dissipation. This is a primary, fundamental reason why the thermodynamic efficiency of isothermal nanomotors can in principle achieve unity in spite of strong dissipation. For example, the thermodynamic efficiency of an F1-ATPase rotary motor can be close to 100% as recent experimental work has demonstrated [15]. For this to happen, the motor must operate most closely to thermal equilibrium in order to avoid net heat losses. One profound lesson from this is that there is no need to minimize friction on the nanoscale. This is a very misleading misconception that continues to plague research on Brownian motors. For example, the so-called dissipationless ratchets are worthless (more on this below). Very efficient motors can work at ambient temperature and arbitrarily strong friction. There is no need to go to deep, quantum cold temperatures, which require a huge energy expenditure to create in a laboratory.

Every thermal bath and its coupling to the particle can be characterized by the bath spectral density


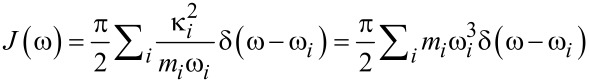


[13–14,16]. It allows η(*t*) to be expressed as


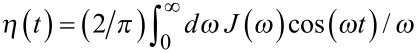


and the noise spectral density via the Wiener–Khinchin theorem, 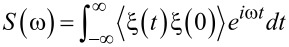, as *S*(ω) = 2*k*_B_*TJ*(ω)/ω. The strict ohmic model, *J*(ω) = ηω, without a frequency cutoff, corresponds to the standard Langevin equation:

[7]
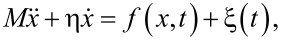


with uncorrelated white Gaussian thermal noise, 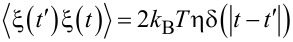. Such noise is singular, and its mean-square amplitude is infinite. This is, of course, a very strong idealization. A frequency cutoff must be physically present, which results in a thermal GLE description with correlated Gaussian noise.

The above derivation can also be straightforwardly repeated for quantum dynamics. This leads to a quantum GLE, which formally looks the same as Equation 3 in the Heisenberg picture with only one difference: The corresponding random force becomes operator-valued with a complex-valued autocorrelation function as shown in Equation 8 [13,16–17].

[8]
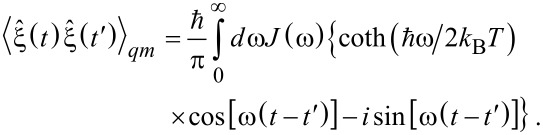


Here, the averaging is done with the equilibrium density operator of the bath oscillators. The classical Kubo result (Equation 6) is restored in the formal limit 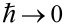. To obtain a quantum generalization of Equation 7, one can introduce a frequency cutoff, *J*(ω) = ηωexp(−ω/ω*_c_*) and split 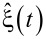 into a sum of zero-point quantum noise, 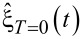, and thermal quantum noise contributions, 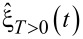, so that 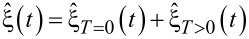. This yields

[9]
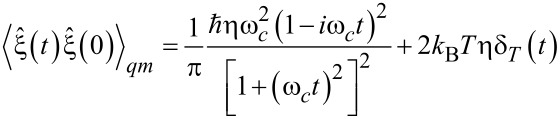


with

[10]
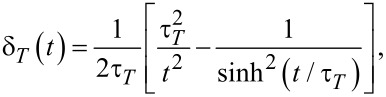


where 
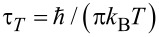
 is the characteristic time of thermal quantum fluctuations. Notice the dramatic change of quantum thermal correlations, from a delta function at 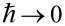, to an algebraic decay 
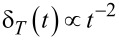
 for finite τ*_T_* and *t >>* τ*_T_*. The total integral of δ*_T_*(*t*) is unity, and the total integral of the real part of the *T* = 0 contribution is zero. In the classical limit, 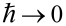, δ*_T_*(*t*) becomes a delta function. Notice also that the real part of the first complex-valued term in Equation 9, which corresponds to zero-point quantum fluctuations, starts from a positive singularity at the origin *t* = 0 in the classical, white noise limit, ω*_c_*→∞, and becomes negative 
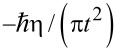
 for *t >* 0. Hence, it lacks a characteristic time scale. However, it cancels precisely the same contribution, but with the opposite sign stemming formally from the thermal part in the limit *t >>* τ*_T_* at *T*≠ 0. Thus, quantum correlations, which correspond to the Stokes or ohmic friction, decay nearly exponentially for ω*_c _>>* 1/τ*_T_*, except for the physically unachievable condition of *T* = 0. Here, we see two profound quantum mechanical features in the quantum operator-valued version of the classical Langevin equation (Equation 7) with memoryless Stokes friction: First, thermal quantum noise is correlated. Second, zero-point quantum noise is present. This is the reason why quantum Brownian motion would not stop even at absolute zero of temperature *T* = 0. A proper treatment of these quantum mechanical features produced a controversial discussion in the literature in the case of nonlinear quantum dynamics when *f*(*x*) is not constant or has a nonlinear dependence on *x*(see [16–17] for further references and details). Indeed, dissipative quantum dynamics cannot be fundamentally Markovian, as already revealed by this short explanation. This is contrary to a popular approach based on the idea of quantum semi-groups, which guarantees a complete positivity of such a dynamics [18]. The main postulate of the corresponding theory (the semi-group property of the evolution operator expressing the Markovian character of evolution) simply cannot be justified on a fundamental level, thinking in terms of interacting particles and fields (a quantum field theory approach). Nevertheless, Lindblad theory and its allies, for example, the stochastic Schroedinger equation [16], are extremely useful in quantum optics where the dissipation strength is very small. The application to condensed matter with appreciably strong dissipation should, however, be done with a great care. This could lead to clearly incorrect results, which contradict exactly solvable models [16]. Nonlinear quantum Langevin dynamics is very tricky, even within a semi-classical treatment, where the dynamics is treated as classical but with colored classical noise corresponding to the real part of 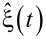 treated as a c-number. As a matter of fact, quantum dissipative dynamics is fundamentally non-Markovian, which is a primary source of all the difficulties and confusion. Exact analytical results are practically absent (except for linear dynamics), and various Markovian approximations to nonlinear non-Markovian dynamics are controversial, being restricted to some parameter domains (e.g., weak system–bath coupling or a weak tunnel coupling/strong system–bath coupling). Moreover, they are susceptible of producing unphysical results (such as violation of the second law of thermodynamics) beyond their validity domains.

Furthermore, a profoundly quantum dynamics has often just a few relevant discrete quantum energy levels, rather than a continuum of quantum states. A two-state quantum system serves as a prominent example. Here, one may prefer a different approach to dissipative quantum dynamics (e.g., the reduced density operator method), leading to quantum kinetic equations for level populations and system coherence [19–22]. This provides a description on the ensemble level and relates to the quantum Langevin equation in a similar manner as the classical Fokker–Planck equation (ensemble description) relates to the classical Langevin equation (description on the level of single trajectories).

#### Minimalist model of a Brownian motor

**Figure 1:**
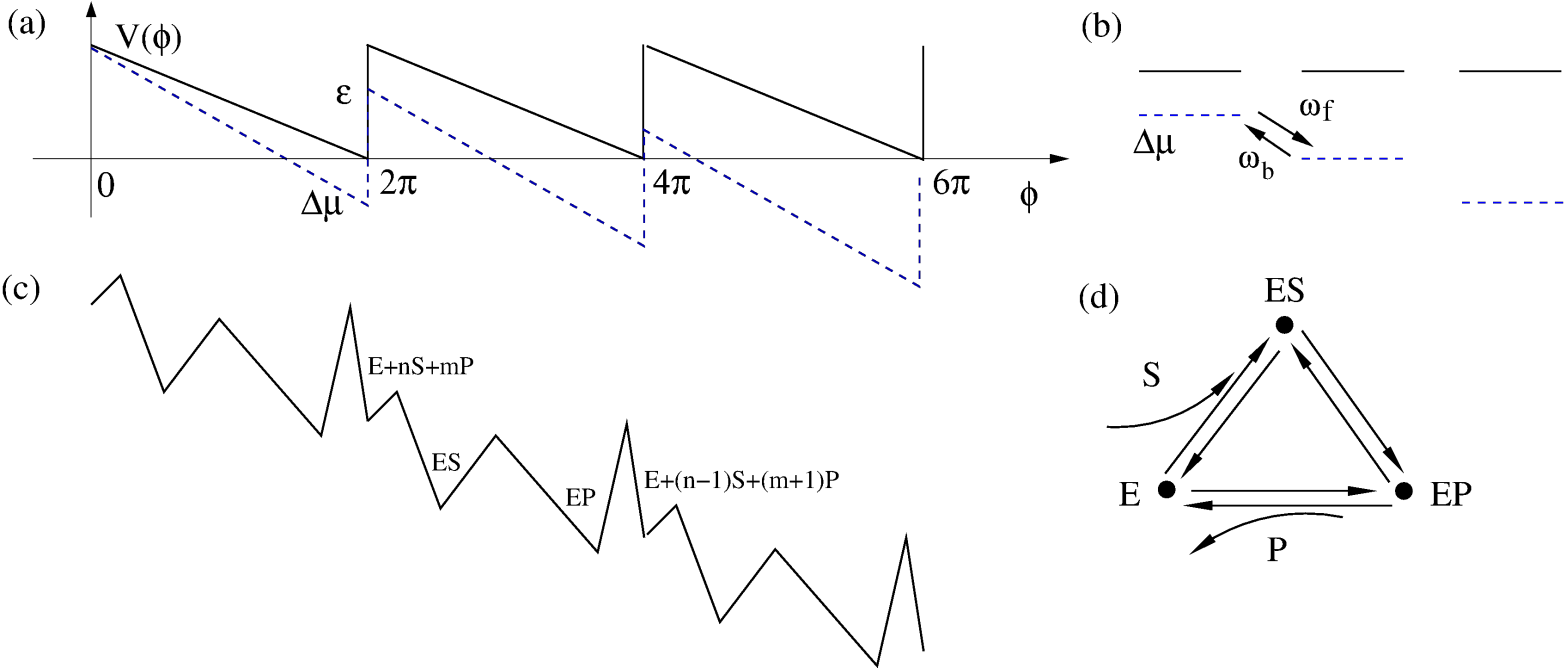
(a) Simplest model for a periodic ratchet potential 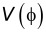 with depth ε. Bias Δμ < 0 per one rotation turn introduces directional rotations. (b) Discrete state model that corresponds to (a) with forward, ω*_f_*, and backward, ω*_b_*, rates calculated, e.g., by solving the Smoluchowski equation, see the text. This picture also holds quantum mechanically with quantum mechanical effects entering the rates in some models, where diagonal and off-diagonal elements of the reduced density matrix are completely decoupled in the energy basis of localized states depicted. (c) The general modeling route is inspired by enzyme dynamics, where an enzyme molecule cycles periodically between a substrate-free state **E**, a state with bound substrate **ES** and a state with bound product **EP**, which correspond to the three metastable states of an enzyme within a continuum of conformational states. Δμ corresponds to the free energy released by transformation *S*→*P* which drives the cyclic rotations of a “catalytic wheel” [24–26], see in (d). This energy can be used to do work against a loading force *f_L_*, which is not shown. For example, an enzyme is an ion pump utilizing the energy of ATP hydrolysis, where ATP is the substrate, and ADP+P_i_ is the product. The useful work done is transfer of an ion across a membrane against the corresponding electrochemical transmembrane gradient.

A minimalist model of a motor can be given by 1D cycling of the motor particle in a periodic potential, 
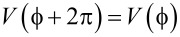
, as shown in Figure 1. This models the periodic turnover of the motor within a continuum of intrinsic, conformational states [3], where 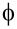 is a chemical cyclic reaction coordinate. The motor cycles can be driven by an energy supplied by a constant driving force or torque, *F*, with free energy Δμ = 2π*F* spent per one motor turn. The motor can perform useful work against an opposing torque or load, *f_L_*, so that the total potential energy is 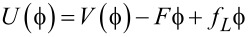. Overdamped Langevin dynamics is described by

[11]
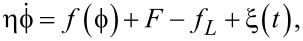


where 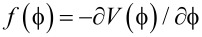, with uncorrelated white Gaussian thermal noise ξ(*t*), 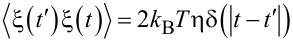. By introducing the stochastic dissipative force 
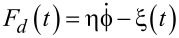
, it can be understood as a force balance equation. The net heat exchange with the environment is 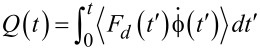
[23], where 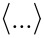 denotes an ensemble average over many trajectory realizations. Furthermore, 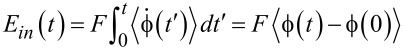 is the energy pumped into the motor turnovers, and 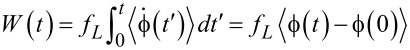 is the useful work done against external torque. The fluctuations of the motor energy 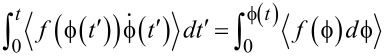 are bounded and can be neglected in the balance of energy in the long run, since *Q*(*t*), *E*_in_(*t*), and *W*(*t*) typically grow linearly (or possibly sublinearly in the case of anomalously slow dynamics with memory, see below) in time. The energy balance yields the first law of thermodynamics: *Q*(*t*) + *W*(*t*) = *E*_in_(*t*). The thermodynamic efficiency is obviously

[12]
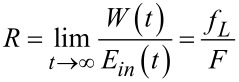


and independent of the potential 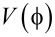. It reaches unity at the stalling force 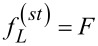. Then, the motor operates infinitesimally slow, 
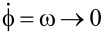
. Henceforth, a major interest present the efficiency *R*_max_ at the maximum of the motor power 
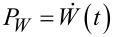
. This one is easy to find in the absence of potential 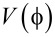, i.e., for *f*(*x*) = 0. Indeed, 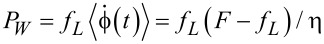. This shows a parabolic dependence on *f_L_* and reaches the maximum at *f_L_* = *F*/2. Therefore, *R*_max_ = 1/2. Given this simple result, many have believed until now that this is a theoretical bound for the efficiency of isothermal motors at maximum power.

**Digression on the role of quantum fluctuations.** Within the simplest model considered (*f*(*x*) = 0) the quantum noise effects do not asymptotically play any role for *T >* 0. This is not generally so, especially within the framework of nonlinear dynamics and at low temperatures where it can be dominant [27]. Most strikingly, the role of the zero-point fluctuations of vacuum (i.e., quantum noise at *T* = 0) is demonstrated in the Casimir effect: Two metallic plates will attract each other in an attempt to minimize the “dark energy” of electromagnetic standing waves (quantized) in the space between the two plates [28]. This effect can be used, in principle, to make a one-shot motor, which extracts energy from zero-point fluctuations of vacuum, or “dark energy” by doing work against an external force, *f_L_*. No violation of the second law of thermodynamics and/or the law of energy conservation occurs because such a “motor” cannot work cyclically. In order to repeatedly extract energy from vacuum fluctuations, one must again separate two plates and invest at least the same amount of energy in this. This example shows, nevertheless, that the role of quantum noise effects can be highly nontrivial, very important, poorly understood, and possibly confusing. And a possibility to utilize “dark energy” to do useful work in a giant, cosmic “one-shot engine” is really intriguing!

#### Thermodynamic efficiency of isothermal engines at maximum power can be larger than one-half

Here it is demonstrated that the belief that *R*_max_ = 1/2 is a theoretical maximum is completely wrong, and in accord with some recent studies [29–32], *R*_max_ can also achieve unity within a nonlinear dynamics regime. For this, we first find stationary 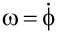 in a biased periodic potential. This can be done by solving the Smoluchowski equation for the probability density *P*(*x*,*t*), which can be written as a continuity equation, 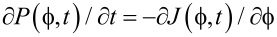, with the probability flux *J*(*x*,*t*) written in the transport form

[13]
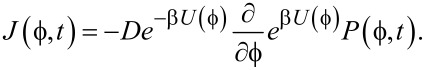


This Smoluchowski equation is an ensemble description and counter-part to the Langevin equation (Equation 11). Here, *D* is the diffusion coefficient related to temperature and viscous friction by the Einstein relation, *D* = *k*_B_*T*/η, and β = 1/*k*_B_*T* is the inverse temperature. For any periodic biased potential, the constant flux, *J* = ω/(2π) = constant, driven by Δμ < 0, as well as the corresponding nonequilibrium steady state distribution, 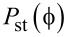, can be found by twice-integrating Equation 13, using 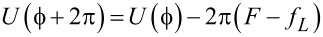 and periodicity of 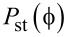. This yields the famous Stratonovich result [33–35] for a steady-state angular velocity of phase rotation

[14]
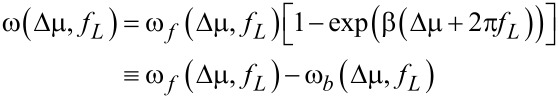


with forward rotation rate

[15]
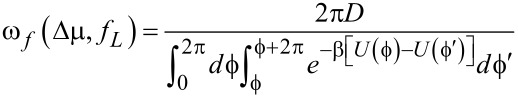


and backward rate ω*_b_*(Δμ,*f_L_*) defined by the second equality in Equation 14. This result is quite general. The motor power is *P_W_*(*f_L_*) = *f_L_*ω*_f_*(Δμ,*f_L_*)[1 − exp(β(Δμ + 2π*f_L_*)) and in order to find *R*_max_ one must find 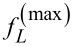 by solving *dP_W_*(*f_L_*)/*df_L_* = 0. Then, 
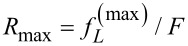
. In fact, Equation 14 is very general. It holds beyond the model of washboard potential, leading to the result in Equation 15. For example, given well-defined potential minima, one can introduce a picture of discrete states with classical Kramers rates for the transitions between those, as described in Figure 1b. Accordingly, within the simplest enzyme model, one has three discrete states. **E** corresponds to an empty enzyme with energy *E*_1_. **ES** corresponds to an enzyme with a substrate molecule bound to it and energy *E*_2_ of the whole complex. **EP** corresponds to an enzyme with product molecule(s) bound to it and energy *E*_3_. The forward cyclic transitions **E**→**ES**→**EP**→**E** are driven by the free energy per one molecule Δμ released in the **S**→**P** transformation facilitated by the enzyme, while the backward cycling, **E**→**EP**→**ES**→**E**, requires backward reaction, **P**→**S**. This is normally neglected in the standard Michaelis–Menthen-type approach to enzyme kinetics as it is very unlikely to occur. This generally cannot be neglected for molecular motors. The simplest possible Arrhenius model for the forward rate of the whole cycle is

[16]
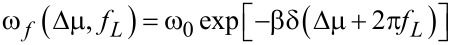


where 0 < δ < 1 describes the asymmetry of the potential drop. Accordingly, the backward rate is ω*_b_*(Δμ,*f_L_*) = ω_0_exp[β(1 −δ) (Δμ + 2π*f_L_*)]. This model allows one to realize under which conditions *R*_max_ can exceed one-half. Here we rephrase a recent treatment in [29–30] and come to the same conclusions. *R*_max_ is a solution of *dP_W_*(*f_L_*)/*df_L_* = 0, which leads to a transcendental equation for *R*_max_

[17]
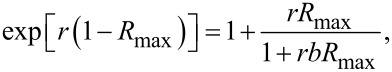


where *r* = |Δμ|/(*k*_B_*T*), *b* = (*k*_B_*T*/2π)∂ln ω*_f_*(Δμ,*f_L_*)/∂*f_L_*. For Equation 16, *b* = −δ. The limiting case *b* = 0 of extreme asymmetry is especially insightful. In this special case, *R*_max_ = [*LW*(*e^1+r^*) − 1]/*r* exactly, where *LW*(*z*) denotes the Lambert W-function. This analytical result shows that *R*_max_→1/2 as *r*→0, while *R*_max_→1 as *r*→∞. Therefore, a popular statement that *R*_max_ is generally bounded by 1/2 is simply wrong. While it is true that in some models this Jacobi bound exists, it is generally not so. Even the simplest model of molecular motors, as considered here by following [29], completely refutes the Jacobi bound as the theoretical limit. Further insight emerges in the perturbative regime, *r <<* 1, which yields in the lowest order of *r*

[18]
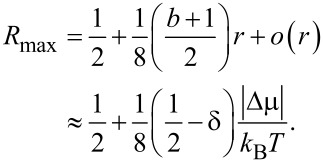


This is essentially the same result as in [30]. Hence, for 0 ≤ δ < 1/2, *R*_max _*>* 1/2 for a small *r*, the effect is small for *r* << 1, but it exists.

The discussed model might seem a bit too crude. However, the result that *R*_max_ can achieve a theoretical limit of unity survives also within a more advanced, yet very simple model. Indeed, let us consider the simplest kind of sawtooth potential (Figure 1) inspired by the above discrete-state model with δ = 0. Then, Equation 15 explicitly yields Equation 19.

[19]
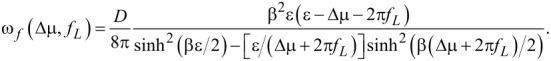


**Figure 2:**
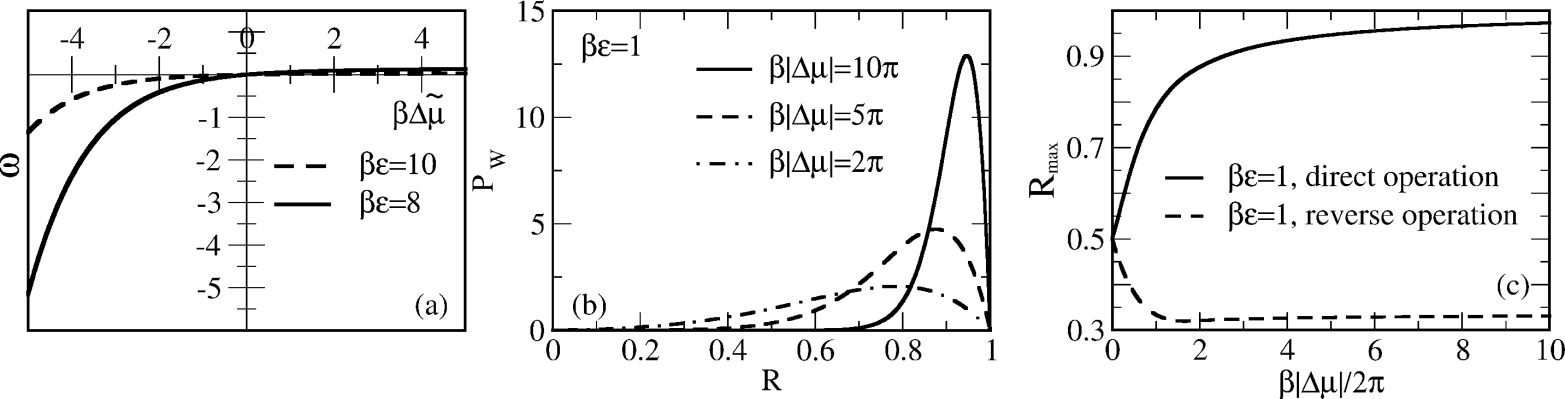
(a) Dependence of the net rotation rate, ω, on the net bias, 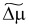, for the most asymmetric sawtooth model depicted in Figure 1a, for two values of the effective barrier height, βε. (b) Dependence of the output power, *P_W_*, on the thermodynamic efficiency, *R*, for βε = 1 and several values of the scaled driving force, β|Δμ|. (c) The maximum power efficiency as a function of driving force for the direct and inverse operation, when the roles of driving force and load are interchanged.

The dependence of ω(Δμ,*f_L_*) on 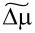:= |Δμ| − 2π*f_L_* is very asymmetric within this model, as shown in Figure 2a.

This is a typical diode-type or rectifier dependence, if the same model is applied to transport of charged particles in a spatially periodic potential, with ω(Δμ,*f_L_*) corresponding to a scaled current and 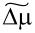 to voltage. Clearly, within the latter context, if an additional, sufficiently slow, periodic voltage signal, *A*cos(ω*t*), is applied at the conditions 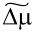 = 0, it will be rectified because of asymmetric *I*–*V* characteristics. This gives rise to a directional, dissipative current in a potential unbiased on average (both spatial and time averages are zero). The effect resulted in a huge amount of literature on rocking Brownian ratchets, in particular, and on Brownian motors, in general as described in a review article [36]. Coming back to the efficiency of molecular motors at maximum power within our model, we see clearly in Figure 2c that it can be well above 1/2, and even close to one. A sharply asymmetric dependence of *P_W_* on *R* = *f_L_*/*F* (Figure 2b) beyond the linear response regime, *P_W_* = 4*P*_max_*R*(1 −*R*), which is not shown therein because of a very small *P*_max_, provides an additional clue on the origin of this remarkable effect. Interestingly, if the work of the motor is reversed, i.e., *f_L_* provides the supply of energy and useful work is done against *F* ≤ *f_L_*, then the motor rotates in the opposite direction on average. This occurs, for example, in such enzymes as F0F1-ATPase [1,3,9], which presents a complex of two rotary motors F0 and F1 connected by a common shaft. The F0 motor uses an electrochemical gradient of protons to rotate the shaft which transmits the torque on the F1 motor. The mechanical torque applied to the F1 motor is used to synthesize ATP out of ADP and the phosphate group, P*_i_*. This enzyme complex primarily utilizes the electrochemical gradient of protons to synthesize ATP. It can, however, also work in reverse and pump protons using the energy of ATP hydrolysis [9]. Moreover, in a separate F1-ATPase motor, the energy of ATP hydrolysis can be used to create mechanical torque and do useful work against an external load, which is experimentally well studied [15]. For the reverse operation, our minimalist motor efficiency becomes 
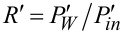
, where 
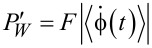
 and 
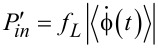
. In this case, 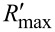 indeed cannot exceed 1/2, as shown in Figure 2c in the lower curve. Such a behavior is also expected from the above discrete-state model, because this corresponds to δ→1 = −*b* in Equation 18. This argumentation can be inverted: If a motor obeys the Jacobi bound, *R*_max_ ≤ 1/2, then it can violate it when working in reverse. Hence, the concept of the Jacobi bound as a fundamental limitation is clearly a dangerous misconception that should be avoided.

#### Minimalist model of a quantum engine

In the quantum case, discrete state models naturally emerge. For example, energy levels depicted in Figure 1b can correspond to the states of a proton pump driven by a nonequilibrium electron flow. This is a minimalist toy model for pumps like the cytochrome c oxidase proton pump [1,37]. The driving force is provided by electron energy, Δμ, released by dissipative tunneling of electrons between donor and acceptor electronic states of the pump. This process is complex. It requires, apart from intramolecular electron transfer, also uptake and release of electrons from two baths of electrons on different sides of a membrane, which can be provided, for example, by mobile electron carriers [1]. However, intramolecular electron transfer (ET) between two heme metalloclusters seems to be a rate limiting step. Such ET presents vibrationally assisted electron tunneling between two localized quantum states [38–39]. Given the weak electron tunneling coupling between the electronic states, the rate can be calculated using the quantum-mechanical Golden Rule. Within the classical approximation of nuclei dynamics (but not that of electrons!), and the simplest possible further approximations, one obtains the celebrated Marcus–Levich–Dogonadze rate,

[20]
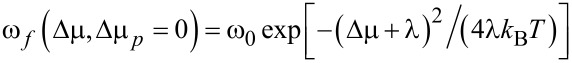


for forward transfer, and ω*_b_*(Δμ,Δμ*_p_* = 0) = ω*_f_*(Δμ,Δμ*_p_* = 0) exp[Δμ/(*k*_B_*T*)]. Here, 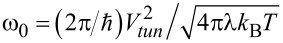 is a quantum prefactor, where *V*_tun_ is the tunneling coupling, and λ is the reorganization energy of the medium. The energy released in the electron transport is used to pump protons against their electrochemical gradient, Δμ*_p_*, which corresponds to 2π*f_L_* within the previous model. Hence, *R* = Δμ*_p_*/|Δμ|. Of course, our model should not be considered as a realistic model for cytochrome c oxidase. However, it allows a possible role of quantum effects to be highlighted that are contained in the dependence of the Marcus–Levich–Dogonadze rates on the energy bias Δμ. Namely, the existence of an inverted ET regime when the rate becomes smaller with a further increase of |Δμ| *>* λ, after reaching a maximum at |Δμ| = λ (activationless regime). The inverted regime is a purely quantum-mechanical feature. It cannot be realized within a classical adiabatic Marcus–Hush regime, for which the rate expression formally appears the same as Equation 20 but with a classical prefactor, ω_0_. Classically, the inverted regime simply makes no physical sense. This fact can be easily realized upon plotting the lower adiabatic curve for the underlying curve crossing problem (within the Born–Oppenheimer approximation), and considering the pertinent activation barriers – the way the Marcus parabolic dependence of the activation energy on the energy bias is derived in textbooks [38]. The fact that the inverted ET regime can be used to pump electrons was first realized within a driven spin–boson model [22,40–42]. The model here is, however, very different, and pumping is not relied on in the inverted ET regime. However, the latter can be used to arrive at a high *R*_max_, close to one. Indeed, within this model, the former (Arrhenius rates) parameter *b* becomes *b* = −1/2 + (|Δμ| − Δμ*_p_*)/(4λ), and Equation 17 is now replaced by

[21]
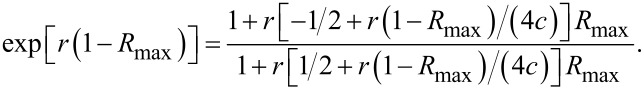


A new control parameter *c* = λ/(*k*_B_*T*) enters this expression. The perturbative solution of Equation 21 for *r* = |Δμ|/*k*_B_*T* << 1 yields

**Figure 3:**
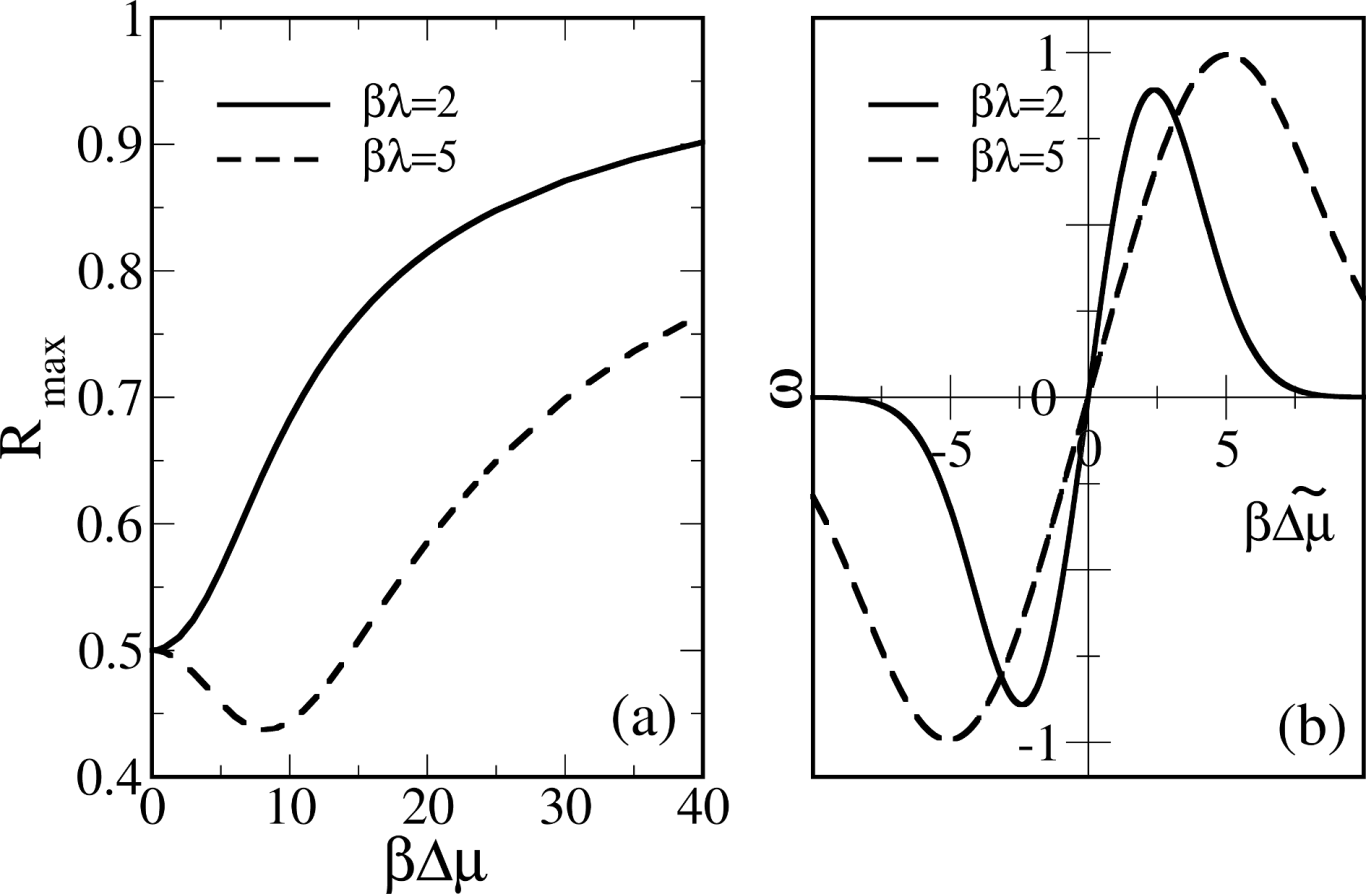
(a) Dependence of *R*_max_ on the absolute value of driving energy Δμ in units of *k*_B_*T* for two values of λ/*k*_B_*T*. Within the perturbative regime, Equation 22 predicts the initial dependence well. (b) Dependence of enzyme velocity ω on 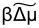. Notice the existence of a maximum ω and negative differential regime.

[22]
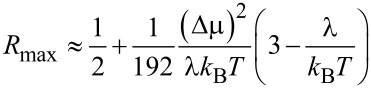


to the lowest second order in |Δμ|/*k*_B_*T* (compare with Equation 18). Hence, *R*_max _*>* 1/2 for λ < 3*k*_B_*T* and *R*_max_ < 1/2 for λ *>* 3*k*_B_*T* in the perturbative regime. However, beyond this, *R*_max_ can essentially be larger than 1/2, as shown in Figure 3a.

These results are also expected for the pump working in reverse when Δμ→−Δμ. Here, we also see a huge difference with the model based on Arrhenius rates. The dependence of the rotation rate, ω, on 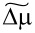 = |Δμ| − Δμ*_p_* is symmetric in this case. However, it exhibits a regime with a negative differential part, where 
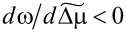
, for 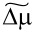 exceeding some critical value that approaches λ for small *T*, as shown in Figure 3b. Here, the reason for the high performance is very different from the case of the asymmetric Arrhenius rates, or asymmetric 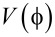. *R*_max_ can be close to one for 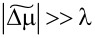. For this to happen, the motor should be driven deeply into the inverted ET regime. Hence, the effect is quantum-mechanical in nature, even if the considered setup looks purely classical. In this respect, the Pauli quantum master equation for the diagonal elements of the reduced density matrix decoupled from the off-diagonal elements has mathematical form of the classical master equation for population probabilities, and the corresponding classical probability description can be safely used. The rates entering this equation can, however, reflect such profound quantum effects as quantum-mechanical tunneling and yield non-Arrhenius dependencies of dissipative tunneling rates on temperature and external forces. The corresponding quantum generalizations of classical results become rather straightforward. The theory of quantum nanomachines with profound quantum coherence effects is, however, still in its infancy.

#### Can a rocking ratchet do useful work without dissipation?

As we just showed, strong dissipation is not an obstacle for either classical or quantum Brownian machines to achieve a theoretical limit of performance. This already indicates that to completely avoid dissipation is neither possible nor desirable to achieve to develop a good nanomachine on the nanoscale. Conversely, the so-called rocking ratchets without dissipation [43–44] are not capable of performing any useful work, despite that they can produce directional transport. However, this directional transport cannot continue against any non-zero force trying to stop it, as will now be demonstrated. The stalling force can become negligibly small, and the thermodynamical efficiency of such a device is zero, very different from genuine ratchets, which must be characterized by a non-zero stalling force [36]. Therefore, a ratchet current without dissipation clearly presents an interesting but futile artefact. The rocking ratchets without dissipation should be named pseudo-ratchets to distinguish them from genuine ratchets characterized by a non-zero stalling force.

Let us consider the following setup. A particle in a periodic potential, *V*(*x*), is driven by a time-periodic force, 
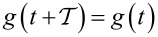
, with period 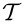. Then, *U*(*x*,*t*) = *V*(*x*) −*xg*(*t*), or *f*(*x*,*t*) = *f*(*x*) + *g*(*t*) in Equation 7. For strong dissipation and overdamped Langevin dynamics, *M*→ 0, the rectification current can emerge in potentials with broken space-inversion symmetry, like one in Figure 1a, under a fully symmetric driving, *g*(*t*) = *A*cos(Ω*t*), 
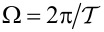
. A broken space-inversion symmetry means that there is no such *x*_0_, so that *V*(−*x*) = *V*(*x* + *x*_0_). Likewise, a periodic driving is symmetric with respect to time reversal if such a *t*_0_ exists (or equivalently, a phase shift 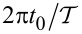), such that *g*(−*t*) = *g*(*t* + *t*_0_). Otherwise it breaks the time-reversal symmetry. Also, higher moments of driving,


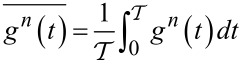


where *n* = 2,3,… are important with respect to a nonlinear response reasoning. The latter moments can also be defined for stochastic driving, using a corresponding time-averaging, with 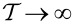. For overdamped dynamics, the rectification current already appears in the lowest second order of 
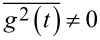
, for a potential with broken spatial-inversion symmetry, and in the lowest third order of 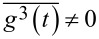 for potentials which are symmetric with respect to inversion *x*→−*x*[36]. These results were easy to anticipate for memoryless dynamics, which displays asymmetric current–force characteristics in the case of an applied static force (broken spatial symmetry), or a symmetric one (unbroken symmetry), respectively. They hold also quantum mechanically in the limit of strong dissipation. The case of weak dissipation is, however, more intricate both classically and quantum mechanically. A symmetry analysis based on the Curie symmetry principle has been developed in order to clarify the issue [36,43]. The harmonic mixing driving [45],

[23]
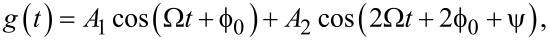


is especially interesting in this respect. Here, ψ is a relative phase of two harmonics, which plays a crucial role. 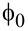 is an absolute initial phase, which physically cannot play any role because it corresponds to a time shift *t*→*t + t*_0_ with 
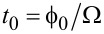
 and hence must be averaged out in the final results, if they are of any physical importance in real world. Harmonic mixing driving provides a nice testbed, because this is the simplest time-periodic driving which can violate the time-reversal symmetry. This occurs for any ψ ≠ 0,π. On the other hand, 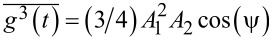. Hence, 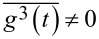, for ψ ≠ π/2,3π/2. Interestingly, 
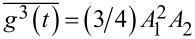
 is maximal for time-reversal symmetric driving. Conversely, 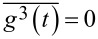, when the time reversal symmetry is maximally broken. Moreover, one can show that all odd moments 
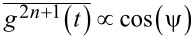
, *n* = 1,2,3,…, vanish for ψ = π/2 or 3π/2. The vanishing of odd moments for a periodic function means that it obeys a symmetry condition 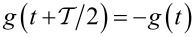. Also, in application to potentials of the form 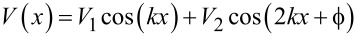, these results mean that 
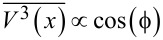
, and 
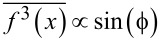
, for the corresponding spatial averages. Hence, for a space-inversion symmetric potential with 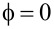, 
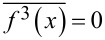
 (also all higher odd moments vanish). Moreover, 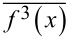 is maximal, when the latter symmetry is maximally broken, 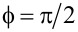. This corresponds to the ratchet potentials. The origin of the rectification current can be understood as a memoryless nonlinear response in the overdamped systems: For 
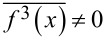
, the current emerges already for standard harmonic driving as a second order response to driving. For 
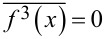
 (e.g., standard cosine potential, *V*_2_ = 0), one needs 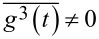 for driving to produce the ratchet effect. For the above harmonic driving, the averaged current 
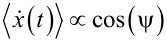
. The same type of response behavior also features a quantum-mechanical, dissipative, single-band, tight-binding model for strong dissipation [46–47]. Very important is that any genuine fluctuating tilt or rocking ratchet is characterized by a non-zero stalling force, which means that the ratchet transport can sustain against a loading force and do useful work against it. It ceases at a critical stalling force. This has important implications. For example, in application to the photovoltaic effect in crystals with broken space-inversion symmetry [36] this means that two opposite surfaces of crystal (orthogonal to current flow) will be gradually charged until the produced photo-voltage stops the ratchet current flow. For a zero stalling force, no steady-state photo-voltage or electromotive force can in principle emerge!

**Figure 4:**
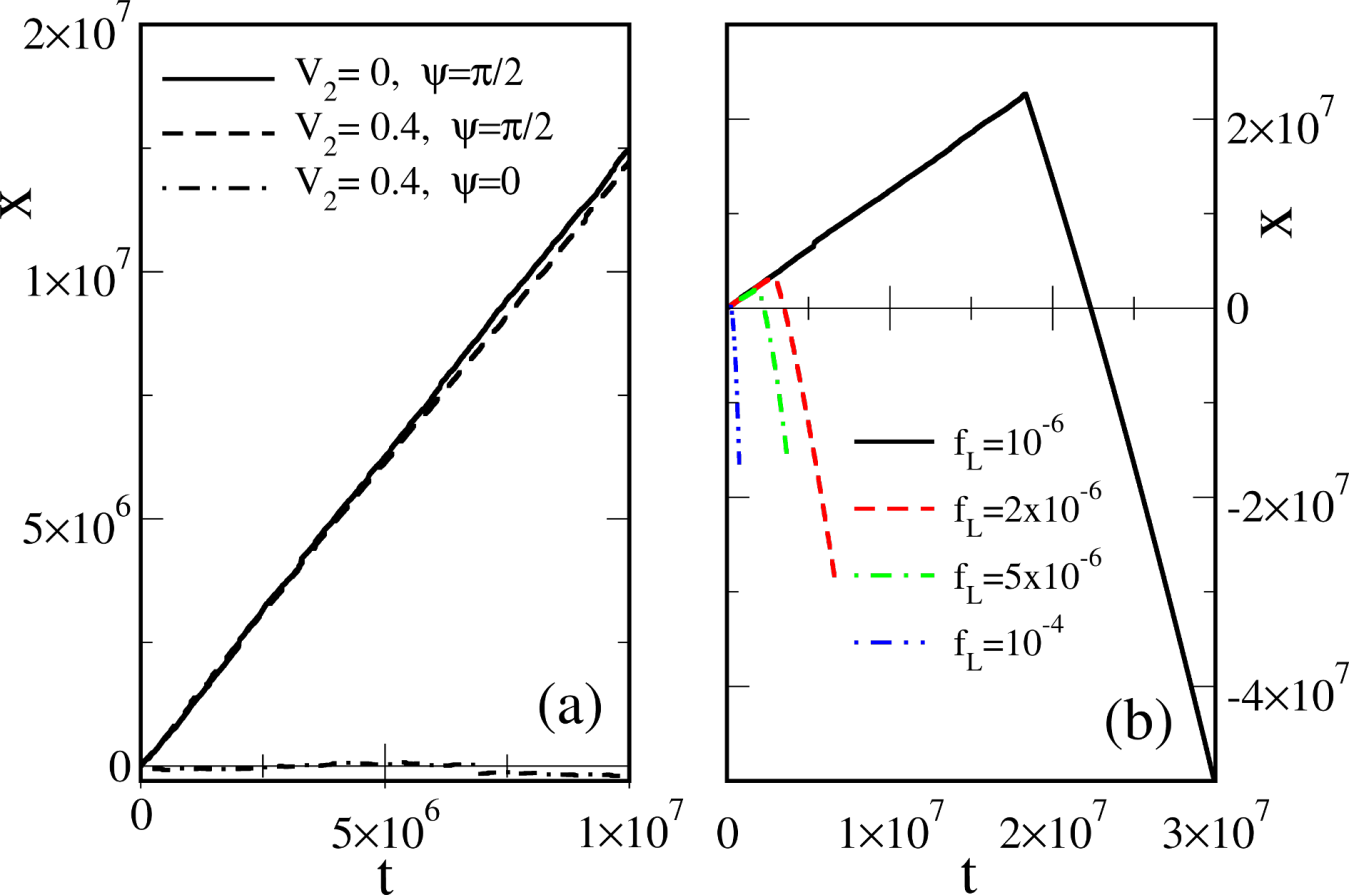
(a) Directed transport in standard cosine potential, *V*_1_ = 1, *V*_2_ = 0, and in a ratchet potential, *V*_1_ = 1, *V*_2_ = 0.4, in the case of harmonic mixing driving that breaks the time-reversal symmetry, ψ = π/2, with amplitudes *A*_1_ = 5, *A*_2_ = 2, and frequency Ω = 1. Transport ceases at ψ = 0 even for ratchet potential with broken symmetry, when the time-reversal symmetry is restored (dash-dotted line). (b) Influence of a tiny (as compare with the periodic force modulation) constant loading force on transport for the case of a ratchet potential in part (a). The transport ceases after some random time, which depends on *f_L_* and initial conditions, and the particle returns accelerating back. A very similar picture also emerges for a cosine potential with *V*_2_ = 0 (not shown). The stalling force is obviously zero. Any genuine ratchet and motor must be characterized by a non-zero stalling force. A symplectic leapfrog/Verlet integration scheme (where no spurious dissipation is introduced by numerics) was used to obtain these results.

In the case of weak dissipation, however, memory effects in the current response become essential. Generally, for classical dynamics, 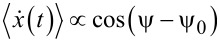, where ψ_0_ is a phase shift which depends on the strength of dissipation with two limiting cases: (i) ψ_0_ = 0 for for overdamped dynamics, and (ii) ψ_0_→π/2 for vanishing dissipation η→0. In the later limit, the system becomes purely dynamical:

[24]
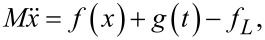


where we added an opposing transport loading force *f_L_*. For example, it corresponds to a counter-directed electrical field in the case of charged particles. Let us consider following [43–44], the two original papers on dissipationless ratchet current in the case of *f_L_* = 0, and the potential *V*(*x*) = −*V*_1_sin(2π*x*)−*V*_2_sin(4π*x*), or *f*(*x*) = *f*_1_cos(2π*x*) + *f*_2_cos(4π*x*), with *f*_1_ = 2π*V*_1_, *f*_2_ = 4π*V*_2_, and driven by *g*(*t*) in Equation 23. The spatial period is set to one and *M* = 1 in dimensionless units. The emergence of a dissipationless current within the considered dynamics has been rationalized within a symmetry analysis in [43], and the subject of directed currents due to broken time–space symmetries has been born. In an immediate follow-up work [44], we have, however, observed that in the above case, the directed current is produced only by breaking the time-reversal symmetry by time-dependent driving, but not otherwise. The breaking of the spatial symmetry of the potential alone does not originate dissipationless current. The current is maximal at ψ = π/2. No current emerges, however, at ψ = 0 even in ratchet potential with broken space-inversion symmetry. Moreover, the presence of a second potential harmonic does not seem to affect the transport at ψ = π/2, as shown in Figure 4a. Here, there are two cases that differ by *V*_2_ = 0, in one case, and *V*_2_≠ 0, in another one.

Moreover, when dissipation is present within the corresponding Langevin dynamics, each and every trajectory remains time-reversal symmetric for ψ = 0. However, for strongly overdamped dynamics, the rectification current in a symmetric cosine potential ceases at ψ = π/2, and not at ψ = 0. Moreover, for an intermediate dissipation, it stops at some ψ_0_, 0 < ψ_0_ < π/2, as shown in [48]. Which symmetry forbids it then, given a particular non-zero dissipation strength? Dynamic symmetry considerations fail to answer such simple questions and are thus not infallible. The symmetry of individual trajectories within a Langevin description simply does not depend on the dissipation strength, which can be easily understood from a well-known dynamical derivation of this equation as presented above. Therefore, a symmetry argumentation based on the symmetry properties of single trajectories is clearly questionable, in general. The spontaneous breaking of symmetry is a well-known fundamental phenomenon both in quantum field theory and the theory of phase transitions. In this respect, any chaotic Hamiltonian dynamics possess the following symmetry: for any positive Lyapunov exponent, there is a negative Lyapunov exponent having the same absolute value of the real part. The time reversal changes the sign of the Lyapunov exponents. This symmetry is spontaneously broken in Hamiltonian dynamics by considering the forward evolution in time [49]. It becomes especially obvious upon coarse-graining, which is not possible to avoid neither in real life nor in numerical experiments. By the same token, the time irreversibility of the Langevin description given time-reversible trajectories is primarily a statistical and not a dynamical effect.

The emergence of such a current without dissipation has been interpreted as a reincarnation of the Maxwell–Loschmidt demon [44], and it has been argued that this demon is killed by a stochastically fluctuating absolute phase 
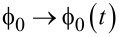
, with the relative phase ψ being fixed. In this respect, even in highly coherent light sources such as lasers, the absolute phase fluctuations cannot be avoided in principle. They yield a finite bandwidth of laser light. The phase shift ψ can be stabilized, but not the absolute phase. The typical dephasing time of semiconductor lasers used in laser pointers is in the range of nanoseconds, whereas in long tube lasers it is improved to milliseconds [50]. This is the reason why some averaging over such fluctuations must always be done (see [35], Chapter 12). The validity of this argumentation has been analytically proven in [44] with an exactly solvable example of a quantum-mechanical, tight binding model driven by harmonic mixing with a dichotomously fluctuating 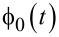. Even more spectacularly, this is seen in dissipationless, tight-binding dynamics driven by an asymmetric, stochastic, two-state field. The current is completely absent even for 
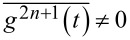
, as an exact solution shows [27]. Hence, dissipation is required to produce a ratchet current under stochastic driving *g*(*t*). The validity of this result is far beyond the particular models in [27,44,46] because any coherent quantum current (one carried by Bloch electron with non-zero quasi-momentum) is killed by quantum decoherence produced by a stochastic field. Any dissipationless quantum current will proceed on a time scale smaller than the decoherence time.

Moreover, it is shown here that the directed transport without dissipation found in [43–44], and the follow-up research cannot do any useful work against an opposing force, *f_L_*. Indeed, the numerical results shown in Figure 4b reveal this clearly: After some random time (which depends, in particular, on the initial conditions and on the *f_L_* load strength), the rectification current ceases. As a matter of fact, the particle then moves back much faster, with acceleration. The smaller the *f_L_*, the longer the directional normal transport regime and smaller back acceleration, and nevertheless the forward transport is absent asymptotically. Therefore, this “Maxwell demon” cannot asymptotically do any useful work, unlike for example, highly efficient ionic pumps – the “Maxwell demons” of living cells working under the condition of strong friction. Plainly said, a dissipationless demon cannot charge a battery, it is futile. Therefore, the consideration of such a device as a “motor” cannot be scientifically justified. It is also clear that with vanishing friction, the thermodynamic efficiency of rocking Brownian motors also vanishes. Therefore, a naive feeling that smaller friction provides higher efficiency is completely wrong, in general.

The following is a brief summary of the major findings of this section. First, friction and noise are intimately related in the microworld, which is nicely seen from a mechanistic derivation of (generalized) Langevin dynamics. It results from hyper-dimensional Hamiltonian dynamics with random initial conditions like in a molecular dynamics approach. For this reason, the thermodynamic efficiency of isothermal nanomotors can reach 100% even under conditions of very strong dissipation, in the overdamped regime where the inertial effects become negligible. Quite on the contrary, thermodynamical efficiency of low-dimensional dissipationless Hamiltonian ratchets is zero. Therefore, they cannot serve as a model for nanomotors in condensed media. Moreover, the geometrical size of some current realizations of Hamiltonian ratchets with optical lattices exceed that of F1-ATPase by several orders of magnitude. In this respect, the readers should be reminded that a typical wavelength of light is about 0.5 μm, which is the reason why motors such as F1-ATPase cannot be seen in a standard light microscope. Hence, the whole subject of Hamiltonian dissipationless ratchets is completely irrelevant for nanomachinery. Second, the thermodynamical efficiency at maximum power in nonlinear regimes can well exceed the upper bound of 50%, which is valid only for a linear dynamics. Therefore, nonlinear effects are generally very important to construct a highly efficient nanomachine. Third, important quantum effects can be already observed within the rate dynamics with quantum rates. For example, these rates can be obtained using a quantum-mechanical perturbation theory in tunnel coupling (within a Fermi’s Golden Rule description) whose particularly simple limit results in Marcus–Levich–Dogonadze rates of nonadiabatic tunneling.

### Adiabatic pumping and beyond

Having realized that thermodynamic efficiency at maximum power can exceed 50%, a natural question emerges: How to arrive at such an efficiency in practice? Intuitively, the highest thermodynamical efficiency of molecular and other nanomotors can be achieved for an adiabatic modulation of potential when the potential is gradually deformed so that its deep minimum gradually moves from one place to another and a particle trapped near this minimum follows adiabatic modulation of the potential in a peristaltic-like motion. The idea is that the relaxation processes are so fast (once again, a sufficiently strong dissipation is required!) that they occur almost instantly on the time scale of potential modulation. In such a way, the particle can be transferred in a highly dissipative environment from one place to another practically without heat losses, and can do useful work against a substantial load (see the discussion in [51]). If at any point in time the motor particle stays near the thermodynamic equilibrium, then in accordance with FDT, the total heat losses to the environment are close to zero. Therefore, thermodynamic efficiency of such an adiabatically operating motor can, in principle, be close to the theoretical maximum. One can imagine, given the three particular examples presented above, that it can be achieved, in principle, at the maximum of power for arbitrarily strong dissipation. The design of the motor thus becomes crucially important. Such an ideal motor can also be completely reversible. However, to arrive at the maximum thermodynamic efficiency at a finite speed is a highly nontrivial matter indeed.

#### Digression on the possibility of an (almost) heatless classical computation

Now, an important digression is considered. In application of these ideas to the physical principles of computation, the above physical considerations mean the following. Bitwise operation (bit “0” corresponds to one location of the potential minimum and bit “1” to another – let us assume that their energies are equal) does not require, in principle, any energy to finally dissipate. It can be stored and reused during adiabatically slow change of potential. Physical computation can, in principle, be heatless, and it can be also completely reversible at arbitrary dissipation. This is the reason why the original version of the Landauer minimum principle allegedly imposed on computation (i.e., there is a minimum of *k*_B_*T*ln2 of energy dissipated per one bit of computation, 0→1, or 1→0 required) was completely wrong. This was recognized by the late Landauer himself [52] after Bennett [53], Fredkin and Toffoli [54] discovered how reversible computation can be done in principle [55]. Another currently popular version of the Landauer principle in formulations where one either needs to spend a minimum of *k*_B_*T*ln2 energy to destroy or erase one bit of information, or a minimum of *k*_B_*T*ln2 heat is released by “burning” one bit of information, is also completely wrong. These two formulations plainly and generally contradict the second law of thermodynamics, which in the differential form states that *dS* ≥ δ*Q*/*T* (i.e., that the increase of entropy, or loss of information, *dI* ≡ −*dS/k*_B_ln2 – a very fundamental equality, or rather tautology of the physical information theory), is equal to or exceeds the heat exchange with the environment in the units of *T*. For an adiabatically isolated system, δ*Q* = 0; hence, *dI* ≤ 0, i.e., entropy can increase and information can diminish spontaneously, without any heat being produced into the surroundings. This is just the second law of thermodynamics rephrased. As a matter of fact, δ*Q* = *|dI|k*_B_*T*ln2 is the maximal (not minimal!) amount of heat which can be produced by “burning” information in the amount of *dI* bits. To create and store one bit of information, one indeed needs to spend at least *k*_B_*T*ln2 of free energy at *T* = constant, but not to destroy or erase it, in principle. Information can be destroyed spontaneously; however, this can take an infinite amount of time. The Landauer principle belongs to common scientific fallacies. However, at the same time, it has established a current hype in the literature. An “economical” reason for this is that the current clock rate of computer processors has not been increased beyond 10 GHz for over one decade because of immense heat production. Plainly said, it is not possible to further cool the processors down to increase their rate, and the energy consumption becomes unreasonable. We eagerly search for a solution to this severe problem. This problem is, however, a problem of the current design of these processors and our present technology, which indeed provides severe thermodynamical limitations [56]. However, it has a little in common with the Landauer principle as heat is currently produced many orders of magnitude above the minimum of the Landauer principle, which should not be taken seriously as a rigorous, theoretical, universally valid bound anyway. Nevertheless, operation at a finite speed is inevitably related to heat loss. The question is, how to minimize this at a maximal speed? This question is clearly beyond a solution in equilibrium thermodynamics, but belongs rather to kinetic theory. The minimum energy requirements are inevitably related to the question of how fast to compute. This presents an open, unsolved problem.

### Minimalist model of adiabatic pump

**Figure 5:**
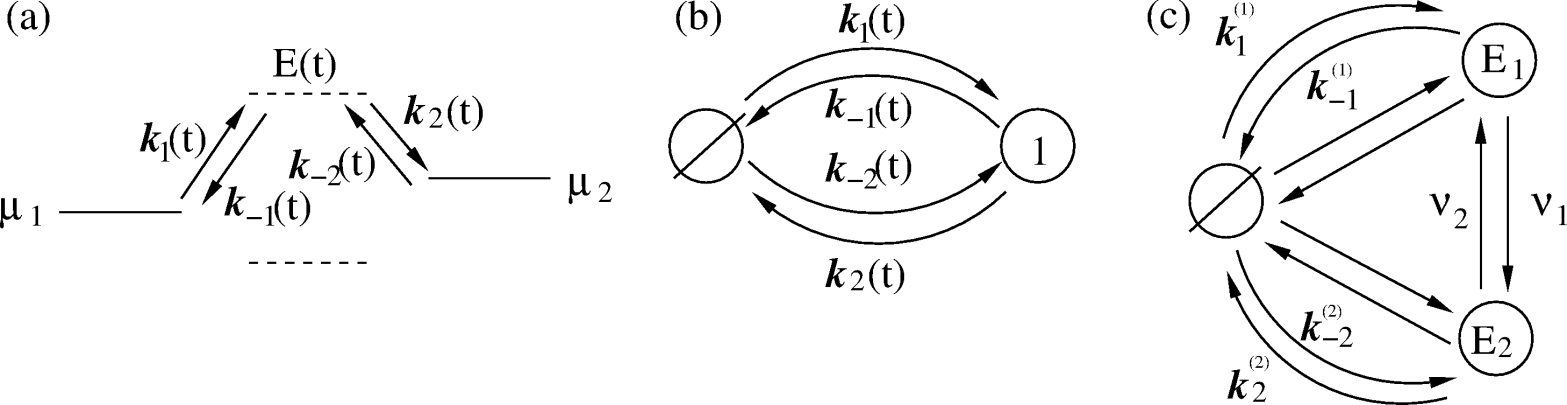
(a) Minimalist model of a pump with one time dependent energy level, *E*(*t*), which can be used to pump particles against a gradient of chemical potential Δμ. (b) Corresponding kinetic scheme with time-dependent rates and two pump states: empty and filled with one transferred particle. Filling and emptying can occur from either particle reservoirs, μ_1_ or μ_2_. (c) Equivalent kinetic scheme corresponding to *E*(*t*), having just two realizations with the transition rates ν_1_ and ν_2_.

Coming back to the adiabatic operation of molecular motors or pumps, a minimalist model based on the time modulation of the energy levels is now analyzed. The physical background of the idea of adiabatic operation is sound. However, can it be realized in popular models characterized by discrete energy levels? The minimalist model contains just one time-dependent energy level, *E*(*t*), and two constant energy levels corresponding to chemical potentials μ_1_ and μ_2_ of two baths of particles between which the transport occurs. They must be considered as electrochemical potentials for charged particles (e.g., Fermi levels of electrons in two leads) or electrochemical potentials of transferred ions in two bath solutions separated by a membrane. Pumping takes place when a time modulation of *E*(*t*) can be used to pump against Δμ = μ_2_ − μ_1 _*>* 0, as shown in Figure 5a. Here, both the energy level *E*(*t*) and the corresponding rates *k*_1_(*t*) and *k*_−1_(*t*), *k*_2_(*t*) and *k*_−2_(*t*) are time dependent. Their proper description would be rate constants, if they were time independent. Given a sufficiently slow modulation and fast equilibration at any instant *t*, one can assume the local equilibrium conditions

[25]
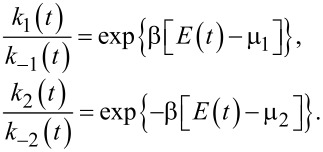


Notice, that this condition is not universally valid. It can be violated by fast fluctuating fields (as shown in [22] and references cited therein) for a plenty of examples and using an approach beyond this restriction within a quantum-mechanical setting. The rates are generally retarded functionals of energy level fluctuations and not functions of instantaneous energy levels. However, a local equilibrium can be a very good approximation. Figure 5b rephrases the transport process in Figure 5a in terms of the states of the pump: empty (state 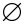) and filled with one transferred particle (state 1). The former state is populated with probability *p*_0_(*t*), and the latter one with probability *p*_1_(*t*), *p*_0_(*t*) + *p*_1_(*t*) = 1. The empty level can be filled with rate *k*_1_(*t*) from the left bath level μ_1_, and with rate *k*_−2_(*t*) from the right bath level μ_2_. The filling flux is thus *j_f_* = (*k*_1_ + *k*_−2_)*p*_0_. Moreover, it can be emptied with rate *k*_2_(*t*) to μ_2_, and with rate *k*_−1_(*t*) to μ_1_. The corresponding master equations reduce to a single relaxation equation because of probability conservation:

[26]
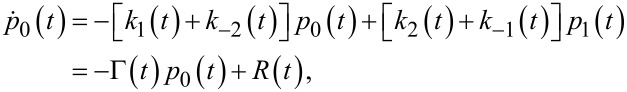


where

[27]
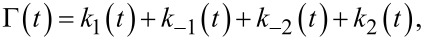


and

[28]
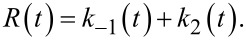


The instantaneous flux between the levels μ_1_ and *E*(*t*) is

[29]
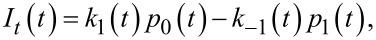


and

[30]
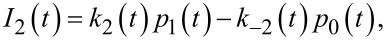


between the levels *E*(*t*) and μ_2_. Clearly, the time averages


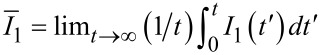


and


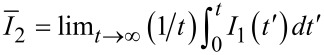


must coincide (
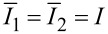
) because particles cannot accumulate on the level *E*(*t*).

First, we show that pumping is impossible within the approximation of a quasi-static rate, that is, when the rates are considered to be constant at a frozen instant in time and one solves the problem within this approximation. Indeed, in this case for a steady-state flux that is an instantaneous function of time we obtain:

[31]
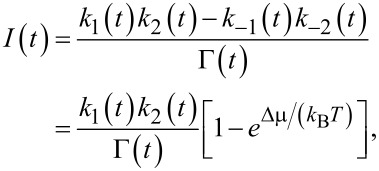


where in the second line, Equation 25 was used. Clearly, for Δμ *>* 0, *I*(*t*) < 0 at any *t*. Averaging over time yields,

[32]
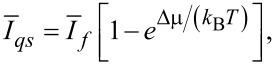


with


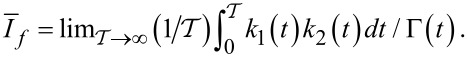


The current always flows from higher μ_2_ to lower μ_1_. The same will happen for any number of intermediate levels *E_i_*(*t*) within such an approximation.

### Origin of pumping

One can, however, easily solve Equation 26 for arbitrary Γ(*t*) and *R*(*t*):

[33]
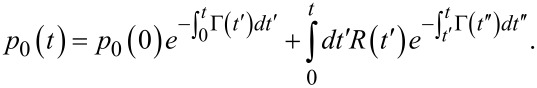


The first term vanishes in the limit *t*→∞ and a formal expression for the steady-state averaged flux, 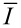, can be readily written as shown in Equation 34 where 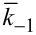 is time-averaged *k*_−1_(*t*).

[34]
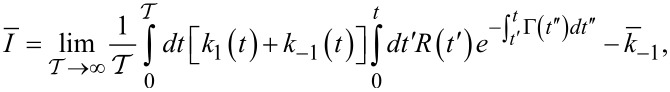


However, to evaluate it for some particular conditions of energy and rate modulation is generally a rather cumbersome task. The fact that pumping is possible is easy to understand, with the following protocol of energy level and rate modulation: (step 1) energy level *E*(*t*) decreases, *E*(*t*) < μ_1_, with an increasing prefactor in *k_±_*_1_(*t*) (left gate opens), and a sharply decreasing prefactor in *k_±_*_2_(*t*) (right gate is closed), a particle enters the pump from the left; (step 2) energy level *E*(*t*) increases, *E*(*t*) *>* μ_2_, and the prefactor in *k_±_*_1_(*t*) sharply drops, the left gate closes and the right one remains closed; (step 3) the right gate opens and the particle leaves to the right; (step 4) the right gate closes, the energy level *E*(*t*) decreases and the left gate opens, so that the initial position in 3D parameter space (two prefactors and one energy level) is repeated, and one cycle is completed. The general idea of an ionic pump with two intermittently opening/closing gates has in fact been suggested a long time ago [57].

Some general results can be obtained within this model for adiabatic slow modulation and related to an adiabatic, geometric, Berry phase, *b*(*t*). The origin of this can be understood per analogy with a similar approach used to solve the Schroedinger equation in quantum mechanics for adiabatically modulated, quasi-stationary energy levels [58], by making the following ansatz to solve Equation 26: *p*_0_(*t*) = *e^ib(t)^R*(*t*)/Γ(*t*) + *c*.*c*. Making a loop in a 2D space of parameters adds or subtracts 2π to *b*(*t*). Furthermore, an additional related contribution, the pumping current, appears in addition to one in Equation 32, with averaging done over one cycle period. This additional contribution is proportional to the cycling rate, ω (see [59] for details). However, it is small and cannot override one in Equation 32 consistently with the adiabatic modulation assumptions. Hence, adiabatic pumping against any substantial bias Δμ *>* 0 is not possible within this model. This indeed can easily be understood by making a sort of adiabatic approximation in Equation 33, 
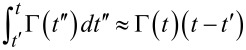
, and doing an integration by parts therein, so that in the long time limit *p*_0_(*t*) ≈ *R*(*t*)/Γ(*t*) + δ*p*_0_(*t*), where 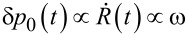. The first term leads to Equation 32, and the second term corresponds to a small perturbative pump current, which vanishes as ω→0. This pump current can be observed only for Δμ = 0, where 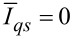. Hence, the thermodynamic efficiency of this pump is close to zero in the adiabatic pumping regime.

Moreover, for realistic molecular pumps, e.g., driven by the energy of ATP hydrolysis, the adiabatic modulation is difficult (if even possible) to realize. A sudden modulation of the energy levels, (e.g., by a power stroke), when the energy levels jump to new discrete positions, is more relevant, especially on a single-molecule level.

### Efficient nonadiabatic pumping

The cases where *E*(*t*) takes on discrete values and is a time-continuous semi-Markovian process can be handled differently. Especially simple is a particular case with *E*(*t*) taking just two values *E*_1_ and *E*_2 _*> E*_1_ with transition rates ν_1_, and ν_2_ between those. Then, the transport scheme in Figure 5b can be rephrased as one in Figure 5c with rate constants 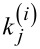 for the transitions to and from the energy levels *E_i_*, *i* = 1,2, *j* = 1,2,−1,−2, and

[35]
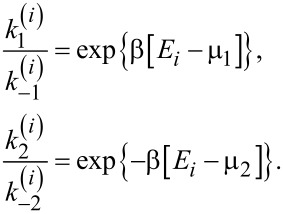


Now we have three populations, *p*_0_ of the empty state, *p*_1_ of level *E*_1_, and *p*_2_ of level *E*_2_. The steady state flux can be calculated as 
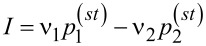
, where 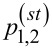 are steady state populations. Straightforward (but somewhat lengthy) calculations yield Equation 36.

[36]
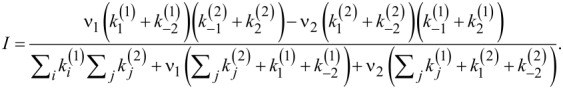


From the structure of this equation it is immediately clear that the flux can be positive for positive Δμ (real pumping) by considering, e.g., the limit: 
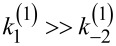
, 
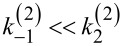
, 
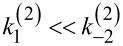
, 
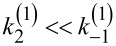
, and ν_1 _*>>* ν_2_. Physically, it is obvious when *E*_1_ < μ_1_, and *E*_2 _*>* μ_2_, together with 
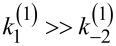
 (i.e., the level *E*_1_ is easily filled from μ_1_, but not from μ_2_ because of a large barrier on the right side – the entrance of pump is practically closed from the right), and 
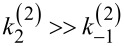
 (i.e., the particle easily goes from *E*_2_ to μ_2_ and cannot go back to μ_1_ because the left entrance is now almost closed). Under these conditions, also 
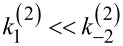
 and 
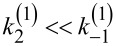
 are well justified. Hence, we obtain for the pumping rate

[37]
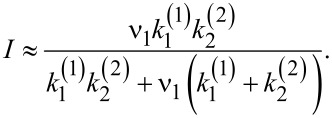


This expression looks like a standard Michaelis–Menthen rate of enzyme operation, which is customly used in biophysics [3] for modeling molecular motors and pumps. The elevation of the *E*(*t*) level from *E*_1_ to *E*_2_ can be effected, e.g., by ATP binding in the case of ionic pumps, with 
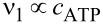
, where *c*_ATP_ is the ATP concentration. This is a simple, basic model for pumps. From Equation 37 it follows that *I* ≈ ν_1_at ν_1_τ << 1, where 
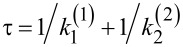
 is the sum of filling and emptying times, and it reaches the maximal pumping rate *I*_max_ ≈ 1/τ, for ν_1_τ *>>* 1. The thermodynamic efficiency of such a pump is *R* = Δμ/Δ*E*, where Δ*E* = *E*_2_−*E*_1_ is the energy invested in pumping. The derivation of the approximation in Equation 37 requires that exp(ε_1,2_/*k*_B_*T*) *>>* 1, where ε_1_ = μ_1_−*E*_1_, and ε_1_ = *E*_2_−μ_2_, which is already well-satisfied for ε_1,2 _*>* 2*k*_B_*T*. Hence, *R* = Δμ/(Δμ + ε_1_ + ε_2_) can be close to one for a large Δμ *>>* ε_1_ + ε_2_. Take for example Δ*E* = 20*k*_B_*T_r_* ≈ 0.5 eV, which corresponds to the typical energy released by ATP hydrolysis. Then, for Δμ = 0.4 eV and ε_1_ = ε_2_ = 2*k*_B_*T_r_* ≈ 0.05 eV, *R* = 0.8. Notice that a typical thermodynamic efficiency of a Na-K pump is about *R* ≈ 0.75. Such a nonadiabatic pumping can be indeed highly thermodynamically efficient with small heat losses. One should note, however, that the question of whether or not the efficiency at the maximum of power, *P_W_* = *I*Δμ, can be larger than one-half or even approach one within this generic model is not that simple. To answer this question, one cannot neglect the backward transport, especially when Δμ becomes close to Δ*E* (*P_W_*(Δμ = Δ*E*) = 0), and a concrete model for the rates must be specified in the exact result (Equation 36). In the case of an electronic pump, like the one used in nature in nitrogenase enzymes, this can be quantum tunneling rates [60], similar to the Marcus–Levich–Dogonadze rate above. Moreover, imposing a very high barrier (intermittent in time) either on the left or right can physically correspond to the interruption of the electron tunneling pathway due to ATP-induced conformational changes, that is, to the modulation of tunnel coupling *V*_tun_(*t*) synchronized with the modulation of *E*(*t*), as occurs in nitrogenase. This question of efficiency at maximum power will be analyzed elsewhere in detail, both for the classical and quantum rate models.

To summarize this section, the concept of the adiabatic operation of molecular machines is sound and should be pursued further. However, the simplest known adiabatic pump operates in fact at nearly zero thermodynamical efficiency, while a power stroke mechanism can be highly efficient within the same model. It seems obvious that in order to realize a thermodynamically efficient adiabatic pumping, the gentle operation of a molecular machine without erratic jumps, a continuum of states is required (or possibly many states depending continuously on an external modulation parameter). Further research is thus highly desirable and necessary.

### How can biological molecular motors operate highly efficiently in highly dissipative, viscoelastic environments?

As it has been clarified above, Brownian motors can work highly efficiently in dissipative environments, causing arbitrarily strong viscous friction acting on a motor. This corresponds to the case of normal diffusion,


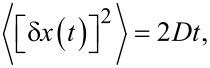


in a force-free case. In a crowded environment of biological cells, diffusion can be, however, anomalously slow,


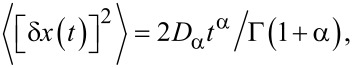


where 0 < α < 1 is the power law exponent of subdiffusion and *D*_α_ is the subdiffusion coefficient [61–62]. There is a huge body of growing experimental evidence for subdiffusion of particles of various sizes, from 2–3 nm (typical for globular proteins) [63–64] to 100–500 nm [65–69] (typical for various endosomes), both in living cells and in crowded polymer and colloidal solutions (complex fluids) physically resembling cytoplasm. There are many theories developed to explain such a behavior [61–62]. One is based on the natural viscoelasticity of such complex liquids (see [70–71] for a review and details), which has a deep dynamical foundation (see above). Viscoelasticity that leads to the above subdiffusion corresponds to a power law memory kernel η(*t*) = η_α_*t*^−α^/Γ(1 −α) in Equation 3 and Equation 6. In this relation, η_α_ is a fractional friction coefficient related to *D*_α_ by the generalized Einstein relation, *D*_α_ = *k*_B_*T*/η_α_. Using the notion of the fractional Caputo derivative, the dissipative term in Equation 3 can be abbreviated as η_α_*d*^α^*x*/*dt*^α^, where the fractional derivative operator *d*^α^*f*(*t*)/*dt*^α^ acting on an arbitrary function *f*(*t*) is just defined by this abbreviation. The corresponding GLE is named the fractional Langevin equation (FLE). Its solution yields the above subdiffusion scaling exactly in the inertialess limit, *M*→0, corresponding precisely to the fractional Brownian motion [70,72], or asymptotically otherwise. The transport in the case of a constant force applied, *f*_0_, is also subdiffusive,


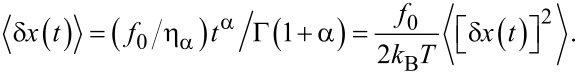


These results correspond exactly to a sub-ohmic model of the spectral density of a thermal bath [16], *J*(ω) = η_α_ω^α^, within the dynamical approach to the generalized Brownian motion. They can be easily understood with an ad hoc Markovian approximation to the memory kernel, which yields a time-dependent viscous friction, 
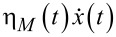
 with 
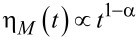
. It diverges, η*_M_*(*t*)→∞, when *t*→∞, which leads to subdiffusion and subtransport within this Markovian approximation. Such an approximation can, however, be very misleading in other aspects [73]. Nevertheless, it provokes the question: How can molecular motors, such as kinesin, work very efficiently in such media characterized by virtually infinite friction, interpolating in fact between simple liquids and solids? It is important to mention that in any fluid-like environment the effective macroscopic friction, 
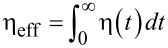
, must be finite. Hence, a memory cutoff time, τ_max_, must exist so that 
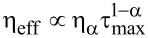
. In real life, τ_max_ can be as large as minutes, or even longer than hours. Hence, on a shorter time scale and on a corresponding spatial mesoscale, it is subdiffusion (characterized by η_α_) that can be physically relevant indeed and not the macroscopic limit of normal diffusion characterized by η_eff_. This observation opens the way for multidimensional Markovian embedding of subdiffusive processes with long range memory upon introduction of a finite number, *N*, of auxiliary stochastic variables. It is based on a Prony series expansion of the power-law memory kernel into a sum of exponentials, 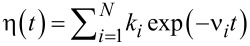, with ν*_i_* = ν_0_/*b^i^*^−1^ and 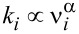. This can be made numerically accurate (which is controlled by the scaling parameter, *b*). Apart from τ_max_ = τ_min_*b^N^*^−1^, it possesses also a short cutoff τ_min_ = 1/ν_0_. The latter naturally emerges in any condensed medium beyond a continuous medium approximation because of its real, atomistic nature. In numerics, it can be made of the order of a time integration step. Hence, it does not even matter within the continuous medium approximation. Even with a moderate *N* ≈ 10–100 (number of auxiliary degrees of freedom), Markovian embedding can be done for any realistic time scale of anomalous diffusion with sufficient accuracy [70,74]. A very efficient numerical approach based on the corresponding Markovian embedding has been developed for subdiffusion in [70,74], and for superdiffusion (α *>* 1) in [75–77]. The idea of Markovian embedding is also very natural from the perspective that any non-Markovian GLE dynamics presents a low-dimensional projection of a hyper-dimensional, singular Markovian process described by dynamical equations of motion with random initial conditions. This fact is immediately clear from a well-known dynamical derivation of GLE, reproduced above. Somewhat surprising is, however, that so few *N* ≈ 10–20 are normally sufficient in practical applications.

The action of a motor on subdiffusing cargo can be simplistically modeled (with the simplest possible theory) by a random force *f*(*t*) alternating its direction when the motor steps on a random network of cytoskeleton [78]. The driven cargo follows a diffusional process 
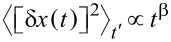
, with some exponent β, which is defined by a trajectory averaging of squared displacements δ*x*(*t*|*t*’) = *x*(*t* + *t*’) −*x*(*t*’) over sliding *t*’. Within such a model, β clearly cannot exceed 2α [79], which corresponds to subtransport with alternating direction in time. Hence, for α < 0.5, cargo superdiffusion (β *>* 1) could not be caused by motors within such a simple approach. However, experiments show [80–81] that freely subdiffusing cargos (e.g., α = 0.4 [80,82]) can superdiffuse when they are driven by motors also for α < 0.5 (e.g., β = 1.3 for α = 0.4 [80])). Therefore, a more appropriate modeling of the transport by molecular motors in viscoelastic environments is required. This was quite recently developed in [83–85], by generalizing the pioneering works on subdiffusive rocking [70,86–89] and flashing [90] ratchets.

**Figure 6:**
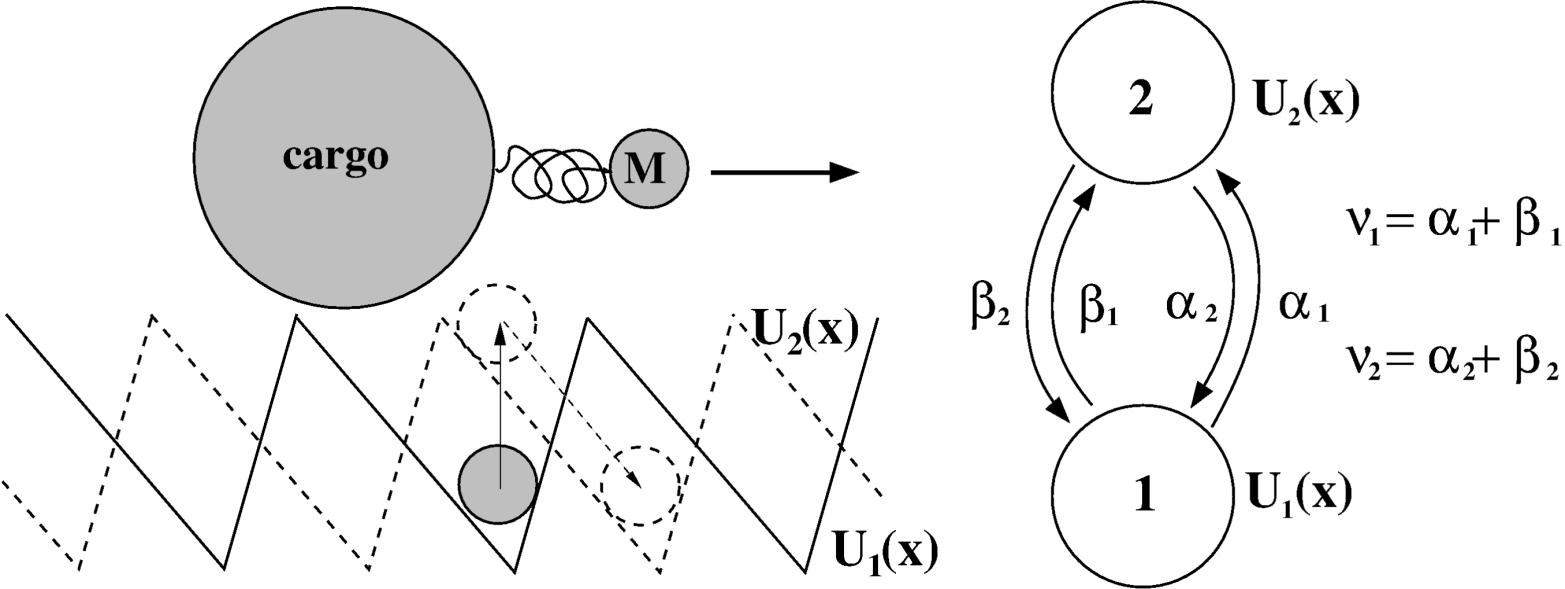
Motor pulling cargo on an elastic linker. The motor can be trapped in a flashing periodic potential (here, two realizations shifted by a half of a spatial period are shown). These fluctuations are caused and driven by conformational fluctuations of the motor protein. The pertinent, minimalist, two-state cyclic model of the corresponding biochemical enzymatic cycle is shown on the right. Mechanical motion, induced by cycling, exerts a back influence on cycling via spatially dependent transition rates. This can cause anomalously slow enzyme kinetics that cannot be characterized by a turnover rate in viscoelastic environments [85].

Viscoelastic effects should be considered on the top of viscous Stokes friction caused by the water component of cytosol. Then, a basic 1D model for a large cargo (20–500 nm) pulled by a much smaller motor (2–10 nm) on an elastic linker (cf., Figure 6) can be formulated as follows [85]

[38]
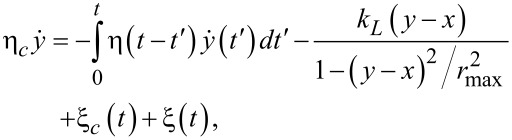


[39]
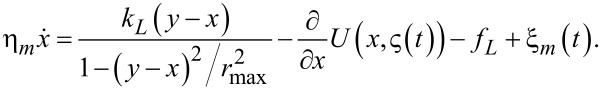


This presents a generalization of a well-known model of molecular motors [91–93] by coupling the motor to a subdiffusing cargo on an elastic linker. Here, both the motor (coordinate *x*) and the cargo (coordinate *y*) are subjected to independent, thermal white noise of the environment, ξ*_m_*(*t*), and ξ*_c_*(*t*), respectively, which obey the corresponding FDRs. Both of the particles are overdamped and characterized by Stokes frictional forces with frictional constants η*_m_*, and η*_c_*. In addition, viscoelastic frictional force acts on the cargo and is characterized by the memory kernel discussed above (fractional friction model) and the corresponding stochastic thermal force, ξ(*t*), with algebraically decaying correlations. It obeys a corresponding FDR. The motor can pull cargo on an elastic linker with spring constant *k_L_* (small extensions) and maximal extension length *r*_max_ (the so-called finite extension nonlinear elastic (FENE) model [94] is used here). The motor (kinesin) is bound to a microtubule and can move along it in a periodic potential, *U*(*x* + *L*,ζ(*t*)) = *U*(*x*,ζ(*t*)), reflecting the microtubule spatial period *L*, and it can do useful work against a loading force, *f_L_*, directed against its motion caused by cyclic conformational fluctuations ζ(*t*). The microtubule is a polar periodic structure with a periodic but asymmetric distribution of positive and negative charges (overall charge is negative) [95]. The kinesin is also charged and its charge fluctuates upon binding negatively charged ATP molecules and dissociation of the products of ATP hydrolysis. This leads to dependence of the binding potential on the conformational variable ζ(*t*). Given two identical heads of kinesin, the minimalist model is to assume that there are only two conformational states of the motor (this is a gross oversimplification, of course) with *U_1,2_*(*x*) := *U*(*x*,ζ*_1,2_*), and *U*_1_(*x* + *L*/2) = *U*_2_(*x*) as an additional symmetry condition, so that a half-step, *L*/2, is associated with conformational fluctuations 1 →2, or 2→1. During one cycle 1→2→1 in the forward direction with the rates α_1_(*x*) and β_2_(*x*), one ATP molecule is hydrolyzed. However, if this cycle is reversed in the backward direction with the rates β_1_(*x*) and α_2_(*x*) (Figure 6), one ATP molecule is synthesized. The dependence of chemical transition rates on the position *x* through the potential *U*_1,2_(*x*) reflects a two-way mechano-chemical coupling. It is able to incorporate allosteric effects, which indeed can be very important for optimal operation of molecular machines [6]. Such effects can possibly emerge, for example, because the probability of binding an ATP molecule (substrate) to a kinesin motor or the release of products can be influenced by the electrostatic potential of the microtubule. In the language of [6], this corresponds to an information ratchet mechanism to distinguish it from the energy ratchet, where the rates of potential switches do not depend on the motor states (no feedback) and are fixed. Such an allostery can be used to create highly efficient molecular machines [6]. In accordance with the general principles of nonequilibrium thermodynamics applied to cyclic kinetics [2],

[40]
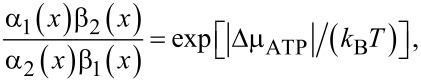


for any *x*, where |Δμ_ATP_| is the free energy released in ATP hydrolysis and used to drive one complete cycle in the forward direction. It can be satisfied, e.g., by choosing

[41]
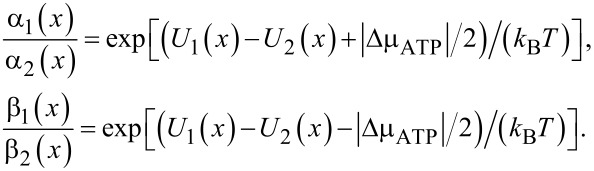


The total rates

[42]
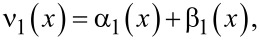


[43]
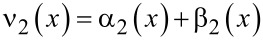


of the transitions between two energy profiles must satisfy

[44]
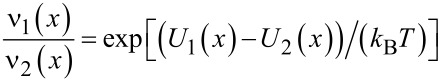


at thermal equilibrium. This is a condition of the thermal detailed balance, where the dissipative fluxes simultaneously vanish both in the transport direction and within the conformational space of a motor [91–92]. It is obviously satisfied for |Δμ_ATP_|→0. Furthermore, on symmetry grounds, not only α*_1,2_*(*x* + *L*) = α*_1,2_*(*x*), β*_1,2_*(*x* + *L*) = β*_1,2_*(*x*), but also, α_1_(*x* + *L*/2) = β_2_(*x*) and α_2_(*x* + *L*/2) = β_1_(*x*). It should be emphasized that linear motors such as kinesin I or II work only one way: they utilize the chemical energy of ATP hydrolysis for doing mechanical work. They cannot operate in reverse on average, i.e., they cannot use mechanical work in order to produce ATP in a long run, even if a two-way mechano-chemical coupling can provide such an opportunity in principle. This is very different from rotary motors such as F0F1-ATPase, which is completely reversible and can operate in two opposite directions. Allosteric effects can also play a role to provide such a directional asymmetry in the case of kinesin motors. Allostery should be considered as generally important for the proper design of various motors best suited for different tasks.

For kinesins, neither cargo nor external force *f_L_* should explicitly influence the chemical rate dependencies on the mechanical coordinate *x*. This leaves still some freedom in the use of different rate models. One possible choice is shown in Equation 45 [85].

[45]
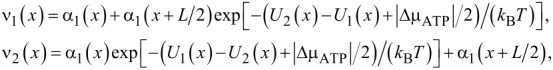


In Equation 45, α_1_(*x*) = α_1_ within the ±δ/2 neighborhood of the minimum of potential *U*_1_(*x*) and is zero otherwise. Correspondingly, the rate β_2_(*x*) = α_1_ relates to the ±δ/2 neighborhood of the minimum of potential *U*_2_(*x*). The rationale behind this choice is that these rates correspond to lump reactions of ATP binding and hydrolysis, and if the amplitude of the binding potential is chosen to be about |Δμ_ATP_|, with a sufficiently large δ, the rates ν_1,2_(*x*) can be made almost independent of the position of the motor along the microtubule [85], when allosteric effects are considered to be almost negligible. This allows for the comparison of this model, featured by bidirectional mechano-chemical coupling, with a corresponding flashing energy ratchet model, where the switching rates between two potential realizations are spatially independent constants, ν_1_ = ν_2_ = α_1_. The latter model has been developed in [84]. Notice that even for reversible F1-ATPase motors, such an energy ratchet model can provide very reasonable and experimentally relevant results [96]. Moreover, if the linker is very rigid, *k_L_*→∞, one can exclude the dynamics of the cargo and consider a one compound particle with a renormalized Stokes friction and the same algebraically decaying memory kernel that moves subdiffusively in a flashing potential. Such an anomalous diffusion molecular motor model has been proposed and investigated in [83]. The main results of [83], which were confirmed and further generalized in [84–85], create the following emerging coherent picture of molecular motors pulling subdiffusing (when free) cargos in viscoelastic environments of living cells. First, if a normally diffusing (when free) motor is coupled to subdiffusing cargo, it will be eventually enslaved by the cargo and also subdiffuse [84]. However, when the motor is bound to a microtubule, it can be guided by the binding potential fluctuations, which are eventually induced by its own cyclic conformation dynamics driven by the free energy released in ATP hydrolysis. It either tends towards a new potential minimum after each potential change, as demonstrated in Figure 6, or can escape by fluctuation to another minimum. A large binding potential amplitude *U*_0 _*>> k*_B_*T* exceeding 10–12*k*_B_*T* (see Figure 6 and the corresponding discussion in [84] to understand why) makes the motor strong. For a large *U*_0_, the probability to escape is small, and the motor will typically slide down to a new minimum and its mechanical motion along the microtubule will be completely synchronized with the potential flashes and conformational cycles. It then steps (stochastically, but unidirectionally) to the right in Figure 6 with mean velocity v = *L*α_1_/2. In such a way, using a power-stroke-like mechanism, a strong motor such as kinesin II (with stalling force 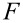 ≈ 6–8 pN) can completely overcome subdiffusion and transport even subdiffusing (when free) cargos very efficiently. This requires, however, that the flashing occurs slower than the relaxation. The larger the cargo, the larger also the fractional friction coefficient η_α_, and the slower the relaxation. The relaxation is algebraically slow. However, it can be sufficiently fast in absolute terms on the time scale 1/α_1_, thus this mechanism is realized for sufficiently small cargos. The results of [83–85] indicate that smaller cargos, 20–100 nm, will typically be transported by strong kinesin motors quite normally, 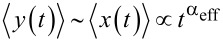, with α_eff_ = 1, at typical motor turnover frequencies ν = α_1_/2 ≈ 1–200 Hz, provided that *f_L_* = 0. This already explains why the diffusional exponent β ≈ 2α_eff_ can be larger than 2α. However, for larger cargos of 100–300 nm, larger turnover frequencies, and when the motor works against a constant loading force *f_L_*, an anomalous transport regime emerges with α ≤ α_eff_ ≤ 1. Clearly, when *f_L_* approaches the stalling force 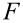, the transport becomes anomalous. The effective transport exponent α_eff_ is thus essentially determined by the binding potential strength, motor operating frequency, cargo size, and loading force, apart from α.

It is very surprising that the thermodynamic efficiency of such a transport can be very high even within the anomalous transport regime. This result is not trivial at all. Indeed, the useful work done by a motor in the anomalous regime against loading force *f_L_* scales sublinearly in time, 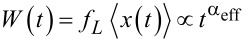 [83,88–89]. However, the free energy transformed into directional motion scales generally as 
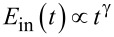
, where 0 < γ ≤ 1. γ = 1 for rocking, or flashing ratchets driven by either by periodic or random two-state force, or by random fluctuations of potential characterized by a well-defined mean turnover rate ν = ν_1_ν_2_/(ν_1_ + ν_2_). Then, 
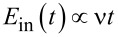
. In the energy balance, the rest, *E*_in_(*t*) − *W*(*t*), is dissipated as a net heat *Q(t)* transferred to the environment. The thermodynamic efficiency is thus [85]

[46]
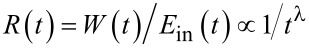


where λ = γ − α_eff_. Hence, 
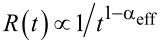
 for γ = 1. It declines algebraically in time, like the mean power 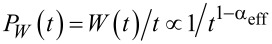. However, temporally, for the typical time required to relocate a cargo within a cell, it can be very high, especially when α_eff_ is close to one. An even more interesting result occurs in the case of bidirectional, mechano-chemical coupling, because the biochemical cycling rates ν_1,2_(*x*) in this case can strongly depend on the mechanical motion for a sufficiently large *U*_0_, when allosteric effects start to play a very profound role. Indeed, if the available |Δμ_ATP_| becomes smaller than the sum of energies required to enhance the potential energy of the motor by two potential flashes (see vertical arrow in Figure 6) during two halves of one cycle, then the enzyme cycling in its conformational space will not generally stop. It can, however, start to occur anomalously slow with a power exponent γ < 1. The average number of forward enzyme turnovers occurring with consumption of ATP molecules scales then as 
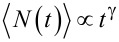
 in time, and 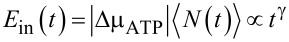. This indeed happens within the model we consider here, see [85] for a particular example with *U*_0_ = 30*k*_B_*T_r_* ≈ 0.75 eV, |Δμ_ATP_| = 20*k*_B_*T_r_* ≈ 0.5 eV, where γ ≈ 0.62 and α_eff_ ≈ 0.556 at the optimal load *f_L_* ≈ 8.5 pN, when the motor pulls a large cargo at the same time. The thermodynamic efficiency declines in this case very slowly, with λ ≈ 0.067, so that *R*(*t*) is still about 70%(!) at the end point of simulations corresponding to a physical time of 3 s. Such a high efficiency is very surprising and provides one more lesson that challenges our intuition and allows us to learn and recognize the power of FDT on the nanoscale. For microscopic and nanoscopic motion occurring at thermal equilibrium, the energy lost in frictional processes is regained from random thermal forces. Therefore, heat losses can, in principle, be small even for an anomalously strong dissipation. This is the reason why the attempts to reduce friction on the nanoscale are misguided. This can, quite counter-intuitively, even hamper efficiency, down to zero as the so-called dissipationless Hamiltonian (pseudo)-motors reveal. One should think differently.

The efficiency at maximum power can also be high in the normal transport operating regime within the discussed model. Indeed, for *U*_0_ = 30*k*_B_*T_r_* and smaller cargo in [85], the transport remains almost normal until the maximum of efficiency is reached at about 80% for an optimal 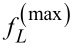 ≈ 9 pN (see Figure 8 in [85], where *f*_0_ corresponds to *f_L_* here). The nearly linear dependence of the efficiency on load until it reaches about 70% indicates that the motor steps with almost the same maximal velocity as at zero loading force *f_L_*. The following simple heuristic considerations can be used to rationalize the numerical results. The motor develops a maximal driving force *F*, which depends on *U*_0_, the motor turnover rate, and temperature (via an entropic contribution), see Figure 6 in [84], where 
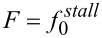
. This is the stalling force. The larger *U*_0_, the stronger the motor and larger *F*. Let us assume that the motor stepping velocity declines from v_0_ to zero with increasing loading force, *f_L_*, as v(*f_L_*) = v_0_[1 − (*f_L_*/*F*)*^a^*], where *a* ≥ 1 is a power-law exponent. Within the linear minimalist model of the motor considered above (and also in an inefficient transport regime within the considered model), *a* = 1, i.e., the motor velocity declines linearly with load. However, in a highly efficient nonlinear regime, this dependence is strongly nonlinear, *a >>* 1. The maximum of the motor power *P_W_*(*f_L_*) = *f_L_*v(*f_L_*) is reached at 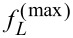 = *F*/(1 + *a*)*^1/a^*, with 
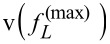
 = *a*v_0_/(1 + *a*). For *a* = 1, 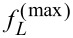 = *F*/2 and the dependence *P_W_*(*f_L_*) is parabolic. With the increase of *U*_0_, *a* is also strongly increased and the dependence *P_W_*(*f_L_*) becomes strongly skewed, in agreement with numerics. Since the input motor power, *P*_in_, does not depend on load within our model in the energy ratchet regimes, the motor efficiency *R* = *P_W_*/*P*_in_ just reflects that of *P_W_*. Hence, the maximum of *R* versus *f_L_* does correspond to the maximum efficiency at maximum power and can exceed 1/2. The same heuristic considerations can be applied to the results presented in [93] for very efficient normal motors. Of course, these results are not necessarily experimentally relevant for, e.g., known kinesin I motors whose maximal efficiency is about 50% [3]. However, our theory can be very relevant for devising artificial motors having other tasks because it provides a biophysically very reasonable model where efficiency at maximum power can be larger than the Jacobi bound of linear stochastic dynamics. It must be stressed, however, that in the anomalous transport regime, one cannot define power and one should introduce the notion of sub-power [88–89].

One should also note the following. Even at *f_L_* = 0, when the thermodynamic efficiency is formally zero, *R*(*t*) = 0, something useful is done: the cargo is transferred over a certain distance by overcoming the dissipative resistance of the environment. However, neither the potential energy of the motor, nor that of the cargo is increased. This is actually normal modus operandi of linear molecular motors like kinesins I or II, very different from ionic pumps whose the primary goal is to increase the (electro)chemical potential of transferred ions (i.e., to charge a battery). Such a *R* = 0 regime, should, however, be contrasted with the zero efficiency of frictionless rocking pseudo-ratchets. In our case, the useful work is done against the environment. Pseudo-ratchets are not capable of doing any useful work in principle.

## Conclusion

In this contribution, some of the main operating principles of minuscule Brownian machines operating on the nano- and microscale are reviewed. Unlike in macroscopic machines, thermal fluctuations and noise play a profound and moreover very constructive role in the microworld. In fact, thermal noise plays the role of a stochastic lubricant, which supplies energy to the Brownian machines to compensate for their frictional losses. This is the very essence of the fluctuation–dissipation theorem: both processes (i.e., frictional losses and energy gain from the thermal noise) are completely compensated on average at thermal equilibrium. Classically, thermal noise vanishes at a temperature of absolute zero (which physically cannot be achieved anyway, in accordance with the third law of thermodynamics), and only then would friction win (classically). However, quantum noise (vacuum zero-point fluctuations) is present even at absolute zero. Therefore, friction cannot win even at absolute zero and quantum Brownian motion never stops. These fundamental facts allow, in principle, for a complete transfer of the driver energy into useful work by isothermal Brownian engines. Their thermodynamic efficiency approaches unity when the net heat losses vanish. This happens when the motor operates close to thermal equilibrium and can be done at any, arbitrarily strong dissipation at ambient temperature. It is not necessary to perform work in the deep quantum cold or to strive for high-quality quantum coherence. A striking example of this is provided by the high transport and thermodynamic efficiency of molecular motors in the subdiffusive transport regime. Operating anomalously slow (in mathematical terms, i.e., exhibiting sublinear dependence of both the transport distance and the number of motor turnovers on time), such motors can be quite fast in absolute terms and can work under a heavy load [85]. In this, and also in other aspects, the intuitive understanding of subdiffusion and subtransport as being extremally slow can be very misleading [97–99]. On the other hand, the frictionless rocking pseudo-ratchets cannot do any useful work, as we clarified in this review.

A scientifically sound possibility to approach the theoretical maximum of thermodynamic efficiency of isothermal motors at arbitrarily strong dissipation and ambient temperatures is intrinsically related to the possibility of reversible dissipative classical computing without heat production. However, such an adiabatic operation would be infinitesimally slow. Clearly, such a motor or computer is not of practical use. Moreover, the adiabatic operation of dissipative pumps involving discrete energy levels is possible only for a vanishing load. Here, a natural question emerges: What is the thermodynamic efficiency at maximum power? The linear dynamics result that *R*_max_ = 1/2 is the theoretical upper bound is, however, completely wrong within nonlinear stochastic dynamics, as shown in this review with three examples. This opens the door for the design of highly efficient Brownian and molecular motors. Moreover, the recent model results in [85] for a normal transport of sufficiently small subdiffusing (when free) cargos by a kinesin motor with a very high thermodynamical efficiency at optimal external load do imply that thermodynamic efficiency at maximum power within that model can also be well above 50%. The earlier results for normal diffusion molecular motors within a very similar model [91–92] obtained in [93] also corroborate such conclusions. Such models are able to mimic allosteric interactions within minimalist model setups. Chemical allosteric interactions, which are intrinsically highly nonlinear, can optimize the performance of various molecular motors. This line of reasoning is especially important for the design of artificial molecular motors [6] and should be pursued further.

Quantum effects are also important to consider to design highly efficient molecular machines, even when quantum coherence does not play any role (that is, on the level of rate dynamics with quantum rates, like in the Pauli master equation). In particular, it has been shown in this review within the simplest model possible that quantum effects (related to the inverted regime of quantum particle transfer) can lead to thermodynamic efficiencies at maximal power larger than one-half for the machine operating both in forward and reverse directions. Quantum coherence could also play a role here, which should be clarified in further research. Undoubtedly, quantum coherence is central for quantum computing, which is obviously reversible [55]. However, this is a different story.

I hope that the readers of this review will find it especially useful in liberating themselves (and possibly others) from some common fallacies, both spoken and unspoken, which unfortunately have pervaded the literature and hinder progress. With this work, a valid, coherent picture emerges.
